# Head-to-head comparison of perfluorobutane contrast-enhanced US and multiparametric MRI for breast cancer: a prospective, multicenter study

**DOI:** 10.1186/s13058-023-01650-3

**Published:** 2023-05-30

**Authors:** Manlin Lang, Ping Liang, Huiming Shen, Hang Li, Ning Yang, Bo Chen, Yixu Chen, Hong Ding, Weiping Yang, Xiaohui Ji, Ping Zhou, ligang Cui, Jiandong Wang, Wentong Xu, Xiuqin Ye, Zhixing Liu, Yu Yang, Tianci Wei, Hui Wang, Yuanyuan Yan, Changjun Wu, Yiyun Wu, Jingwen Shi, Yaxi Wang, Xiuxia Fang, Ran li, Jie Yu

**Affiliations:** 1 Department of Interventional Ultrasound, Fifth Medical Center of Chinese PLA General Hospital & Chinese PLA Medical School, Beijing, 100039 China; 2 Department of Ultrasound, Zhongda Hospital Southeast University, Nanjing, 210009 China; 3 Department of Breast Surgery, Affiliated Hospital of Putian University, Putian, 351100 China; 4 Department of Ultrasound, Xingcheng People’s Hospital, Xingcheng, 125100 China; 5 Department of Ultrasound Medicine, Lu’an People’s Hospital of Anhui Province, Liuan, 237000 China; 6 Department of Ultrasound, The Fifth People’s Hospital of Chengdu, Chengdu, 611130 China; 7 Department of Ultrasound, Huashan Hospital, Shanghai, 200040 China; 8 Department of Ultrasound, Guangxi Medical University Cancer Hospital, Nanning, 530021 China; 9 Department of Ultrasound, The Fourth Hospital of Hebei Medical University, Shijiazhuang, 050011 China; 10 Department of Ultrasound, The Third Xiangya Hospital, Changsha, 410000 China; 11 Department of Ultrasound, Peking University Third Hospital, Beijing, 100191 China; 12 General Surgery, Chinese PLA General Hospital, Beijing, 100853 China; 13 Department of Ultrasound, First Affiliated Hospital of Southern University of Science and Technology, Second Clinical College of Jinan University, Shenzhen Medical Ultrasound Engineering Center, Shenzhen People’s Hospital, Shenzhen, 518020 China; 14 Department of Ultrasound Medicine, The First Affiliated Hospital of Nanchang University, Nanchang, 330006 China; 15 Department of Ultrasound, Beijing Friendship Hospital, Beijing, 100050 China; 16 Department of Ultrasound, The 2nd Affiliated Hospital of Harbin, Harbin, 150001 China; 17 Department of Ultrasound, China-Japan Union Hospital of Jilin University, Changchun, 130033 China; 18 Department of Ultrasound, Zhengzhou Central Hospital, Zhengzhou, 450000 China; 19 Department of Ultrasonography, The First Affiliated Hospital of Harbin Medical University, Harbin, 150001 China; 20 Department of Ultrasound, Affiliated Hospital of Nanjing University of Chinese Medicine, Nanjing, 210029 China; 21 Department of Ultrasound, Shengjing Hospital of China Medical University, Shenyang, 110004 China; 22 Department of Ultrasound, The Affiliated Hospital of Inner Mongolia Medical University, Hohhot, 010050 China; 23 Department of Ultrasound, The First Affiliated Hospital of Xinxiang Medical University, Xinxiang, 453100 China; 24 Department of Interventional Ultrasound, Fifth Medical Center of Chinese PLA General Hospital, Beijing, 100039 China

**Keywords:** PFB-CEUS, MP-MRI, Hybrid, Diagnostic model, Breast lesions, Diagnostic performance

## Abstract

**Background:**

Multiparametric magnetic resonance imaging (MP-MRI) has high sensitivity for diagnosing breast cancers but cannot always be used as a routine diagnostic tool. The present study aimed to evaluate whether the diagnostic performance of perfluorobutane (PFB) contrast-enhanced ultrasound (CEUS) is similar to that of MP-MRI in breast cancer and whether combining the two methods would enhance diagnostic efficiency.

**Patients and methods:**

This was a head-to-head, prospective, multicenter study. Patients with breast lesions diagnosed by US as Breast Imaging Reporting and Data System (BI-RADS) categories 3, 4, and 5 underwent both PFB-CEUS and MP-MRI scans. On-site operators and three reviewers categorized the BI-RADS of all lesions on two images. Logistic-bootstrap 1000-sample analysis and cross-validation were used to construct PFB-CEUS, MP-MRI, and hybrid (PFB-CEUS + MP-MRI) models to distinguish breast lesions.

**Results:**

In total, 179 women with 186 breast lesions were evaluated from 17 centers in China. The area under the receiver operating characteristic curve (AUC) for the PFB-CEUS model to diagnose breast cancer (0.89; 95% confidence interval [CI] 0.74, 0.97) was similar to that of the MP-MRI model (0.89; 95% CI 0.73, 0.97) (*P* = 0.85). The AUC of the hybrid model (0.92, 95% CI 0.77, 0.98) did not show a statistical advantage over the PFB-CEUS and MP-MRI models (*P* = 0.29 and 0.40, respectively). However, 90.3% false-positive and 66.7% false-negative results of PFB-CEUS radiologists and 90.5% false-positive and 42.8% false-negative results of MP-MRI radiologists could be corrected by the hybrid model. Three dynamic nomograms of PFB-CEUS, MP-MRI and hybrid models to diagnose breast cancer are freely available online.

**Conclusions:**

PFB-CEUS can be used in the differential diagnosis of breast cancer with comparable performance to MP-MRI and with less time consumption. Using PFB-CEUS and MP-MRI as joint diagnostics could further strengthen the diagnostic ability.

*Trial registration* Clinicaltrials.gov; NCT04657328. Registered 26 September 2020.

*IRB number* 2020-300 was approved in Chinese PLA General Hospital. Every patient signed a written informed consent form in each center.

**Supplementary Information:**

The online version contains supplementary material available at 10.1186/s13058-023-01650-3.

## Introduction

There are more than twenty histological classifications for breast lesions. Diagnostic imaging for the identification of lesion features remains challenging due to the overlapping characteristics of some benign and malignant lesions [1, 2]. It is essential that imaging performance be optimized to meet the needs of patients with breast lesions.

Blood perfusion is one of the vital indicators in differentiating breast lesions [3]. MP-MRI and CEUS are imaging methods that provide visualization of tumor blood perfusion [4]. Initially, studies suggested that multiparametric MRI (MP-MRI) is more accurate than conventional ultrasound (US) or mammography [5, 6] and could improve DCE-MRI specificity [7]. However, precautions must be taken in patients with renal dysfunction with respect to the use of MRI contrast agents [8]. Cost, the timing of the MRI exam, claustrophobia, and patients with morbid obesity who cannot be accommodated within the MRI bore are clinically relevant and commonly considered limitations of MRI [9].

Contrast-enhanced ultrasound (CEUS) is a nonirradiating, accessible, and easy-to-implement imaging technique that is a powerful supplementary problem-solving tool in the context of MRI [10]. However, CEUS is not always recommended for routine clinical diagnostic use by several breast lesion management guidelines, such as the American Cancer Society (ACS) [11], National Comprehensive Cancer Network (NCCN) [6], or the European Federation of Societies for Ultrasound in Medicine and Biology (EFSUMB) [12]. The main reason cited is the lack of a specific pattern indicating malignancy because several retrospective studies have shown inconsistent results (Additional file 1: Table S1) [13–17].

Currently, sulfur hexafluoride (SHF) is the most widely available CEUS agent worldwide but has inconsistent diagnostic results. Image quality in the detection of breast cancer using SHF-CEUS is limited by insufficient signal generation from microbubbles released at high frequencies from linear transducers [18]. Upon reconstitution with sterile water, stabilized microspheres of perfluorobutane (PFB), which are stabilized by a stable outer shell with hydrogenated egg phosphatidylserine, can resist acoustic pressure, leading to increased microbubble oscillations that minimize collapse and loss of signal [19–21]. Additionally, safety of PFB in the diagnosis of breast lesions has been confirmed in a prospective open-label multicenter phase 3 study [14]. Therefore, PFB demonstrates theoretical potential to improve the quality of breast CEUS examination with sufficient safety for patients.

Therefore, we explored the application of feature analysis in a multicenter and larger patient cohort by comparing head-to-head PFB-CEUS and MP-MRI to investigate the difference in diagnostic ability between PFB-CEUS and MP-MRI for the identification of breast cancer and whether the combination of PFB-CEUS can improve the MP-MRI diagnostic capacity of breast cancer.

## Materials and methods

### Study design

**Fig. 1:**
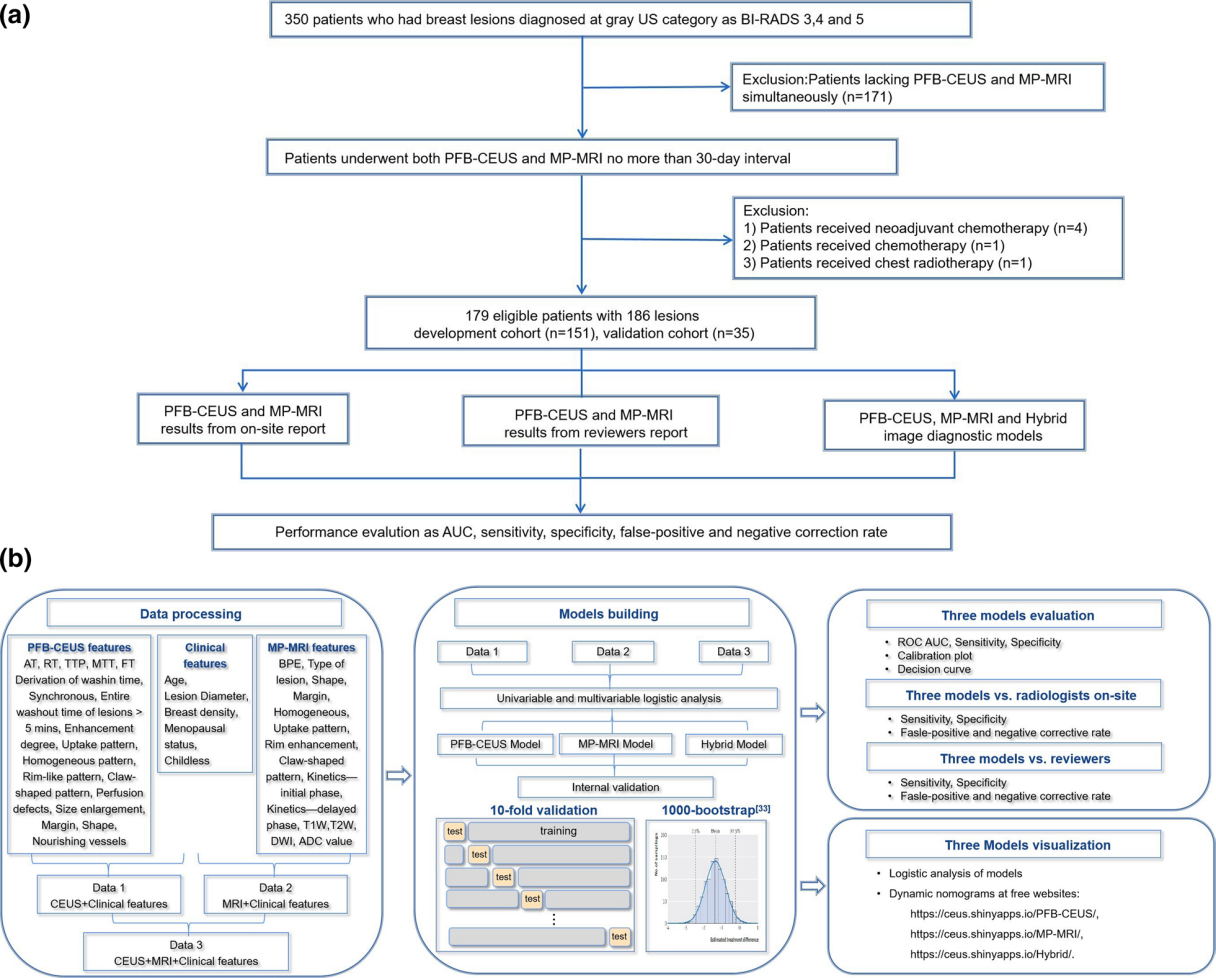
Diagram of **a** patient selection and **b** model construction. *PFB-CEUS* perfluorobutane contrast-enhanced ultrasound, *MP-MRI* multiparametric magnetic resonance imaging

This study was a prospective, multicenter trial (ClinicalTrials.gov: NCT04657328) in which study participants were consecutively recruited from 17 centers in China from September 2020 to February 2021 (Additional file 1: Table S2). The inclusion criteria and exclusion criteria are shown in flowchart Fig. 1 and Additional file 1: Supplementary Materials and Methods S1. Twenty percent of the subjects were randomly selected to form a validation cohort, and the remaining 80% were used as a development cohort. Institutional review board and regulatory approval were granted by the Chinese PLA General, and all the patients provided written informed consent. All the investigators and authors had complete access to all the study results, and the authors had full control of the data and statistical results included in this report.

### Data collection

Clinical characteristics and imaging features of PFB-CEUS and MP-MRI were prospectively collected in a Research Electronic Data Capture (REDCap) database.

### Imaging protocol

A total of 25 and 27 radiologists with 5–10 years of experience performed the PFB-CEUS examination and MP-MRI examination, respectively. PFB-CEUS and MP-MRI images from the 17 centers were stored in DICOM data format.

#### PFB-CEUS

The PFB-CEUS examinations were performed using 15 devices (Additional file 1: Table S3). With the linear probe, pulse inversion harmonic imaging and a mechanical index of 0.18–0.24 were used for PFB-CEUS. A bolus injection of 0.015 ml/kg perfluorobutane-filled microbubble contrast agent (Sonazoid; GE Healthcare, Oslo, Norway) was administered via a ≧ 22-gauge catheter line placed in the antecubital vein. A 5-mL flush of 0.9% sodium chloride solution was administered after injection of the contrast agent. The dispersion was prepared just prior to use and administered within 2 h of preparation. The imaging timer was started simultaneous to the completion of the contrast agent injection with continuous assessment of the lesions for 1 min, followed by intermittent scanning for 10 s at the following time points: 1 min and 30 s, 2 min, 3 min, 4 min, and 5 min. Both the mass of interest and the breast involved were evaluated, and patients with multiple lesions were included based on the interval time between two injections being 20 min.

#### MP-MRI

The MP-MRI examinations were performed using eight devices (Additional file 1: Table S4) and performed on 1.5 or 3.0 Tesla systems using a dedicated bilateral breast coil. All protocols followed ACR BI-RADS Magnetic Resonance Imaging and EUSOMA recommendations [22, 23], which included a T2-weighted sequence and a T1-weighted series acquired before and after the injection of a gadolinium-based contrast agent. In all centers, regions of interest (ROI) (typically 3 × 3 × 1 voxels) in the lesion were used to measure the time-signal intensity curves, and ADC maps used for the evaluation were generated by inline monoexponential fitting of the highest and lowest b-value data by the scanner software. A typical MRI exam occupies the MRI system for up to 40 min.

### Imaging analysis

A panel of six radiologists (with each radiologist having ≥ 15 years of experience in breast lesion diagnosis) reviewed 50 cases of PFB-CEUS and MP-MRI images to identify and define the imaging characteristics. The MP-MRI imaging characteristics were identified by combining the panel of six radiologist results with ACR MRI BI-RADS guideline items. Subsequently, for each imaging modality, two radiologists, each with > 5 years of experience, were trained with a ≥ 0.75 kappa value in all the images. The readers were blinded to current and previous breast imaging and histological findings. Discrepancies were resolved by consensus and consultation with one of the radiologists with 20 years of experience.

Quantitative PFB-CEUS parameters of the lesions were acquired using quantitative analysis software, i.e., NovoUltrasound Kit (Precision Health Institute, GE Healthcare China). ROI 1 included the entire tumor boundary on PFB-CEUS imaging while avoiding the surrounding parenchyma. ROI 2 encircled the normal-appearing parenchyma, including the same image acquisition plane as far as possible from the breast tumor [24] (Additional file 1: Figure S1).

## Reference standard

For all of the lesions, histopathology was used as the reference standard. Tissue samples of the lesion were obtained by US-guided biopsy or surgical resection. Biopsy was performed using a 14/16-gauge core needle with real-time US/PFB-CEUS guidance. Specimens were reviewed by two senior pathologists from each academic practice.

### Statistical analysis

To detect a difference of 0.1 between a diagnostic test using PFB-CEUS, with an area under the ROC curve (AUC) of 0.85, and using MRI, with an AUC of 0.95, a sample size of 81 malignant and 55 benign lesions was needed to achieve 90% power at a significance level of 0.05. Sample size calculations were performed using PASS version 11 (NCSS, LLC, Kaysville, Utah, USA).

Univariate and multivariable logistic regression analyses were performed to select the risk factors for breast cancer diagnosis and construct three different models: the PFB-CEUS model, MP-MRI model and hybrid model. Each model contained clinical features (age, menopausal status and nulliparity) and radiological features.

Radiological intranodular features of PFB-CEUS included the diameter of lesions, fall time (FT), rise time (RT), time-to-peak (TTP), mean transit time (mTT), arrival time (AT), derivation of washing time (earlier, later, or synchronous), degree of enhancement (hyperenhancement, isoenhancement, or hypoenhancement), uptake pattern (centripetal, centrifugal, diffuse, or no enhancement), presence or absence of entire washout time > 5 min, heterogeneous pattern, rim-like enhancement, claw-shaped pattern, perfusion defects, size enlargement, noncircumscribed margin, and irregular shape. Perinodular features of PFB-CEUS contained nourishing vessels and breast density. The diameter of lesions was defined as the maximum tumor diameter measured on conventional grayscale US. Radiological intranodularn features of MP-MRI included the diameter of lesions, pattern (noncircumscribed margin, irregular shape, homogeneity, etc.), type of lesion (focus/foci only, mass, or nonmass enhancement), uptake pattern (centripetal, centrifugal, diffuse, or other), kinetics—initial phase (slow, medium, or rapid), kinetics—delayed phase (persistent, plateau, or washout), signal intensity of T1 and T2, (high, equal, or low), DWI (high or low), and ADC value according to the Breast Imaging Reporting and Data System MRI lexicon Perinodular features of MP-MRI contained breast density and background parenchymal enhancement (minimal, mild, moderate, or marked).

The radiological features of the hybrid model contained all the radiological features of PFB-CEUS and MP-MRI.

Model selection was performed under stepwise criteria when necessary. Bootstraps of 1000 resamples and fivefold and tenfold cross-validation were performed to evaluate the performance of the models (the bootstrap process is shown in Additional file 1: Supplementary Materials and Methods S2) [25]. Nomograms used are freely available online. Each of the websites was https://ceus.shinyapps.io/PFB-CEUS for the PFB-CEUS model, https://ceus.shinyapps.io/MP-MRI/for the MP-MRI model, and https://ceus.shinyapps.io/Hybrid/ for the hybrid model. Discrimination was quantified by using the area under the receiver operating characteristic curve (AUC), sensitivity, and specificity. Calibration was assessed with the Hosmer‒Lemeshow test and calibration plot. Descriptive analysis summarized the patient characteristics. The operating point selection method is shown in Additional file 1: Supplementary Materials and Methods S3. All of the lesions were collected and analyzed as follows:

BI-RADS 4A+ means that in this mode, the lesion is categorized as 4A as a cutoff to consider a malignant lesion, and BI-RADS 3 was categorized as a benign lesion. The plus mark (BI-RASD 4B+ or 4C+) indicates higher categories as the malignant cutoff value. 4A+ means that the malignancy is considered category 4A to 5; 4B+ means that the malignancy is considered category 4B to 5; and 4C+ means that the malignancy is considered category 4C to 5.

According to the definition in previous studies [26], the CEUS BI-RADS score was determined (Additional file 1: Supplementary Materials and Methods S4). MRI BI-RADS results were obtained according to the ACR BI-RADS Atlas [22]. The discriminatory performance of our newly established models was also compared to that of Luo et al. [27] 5-point scoring system (Luo model), Chen et al. [28] benign and malignant 6-pattern model (Chen model), and Yukio et al. [14] benign and malignant 2-pattern model (Yukio model). All analyses were performed using R and Stata (version 15). *P* values less than 0.05 were considered statistically significant.

## Results

### Participant and imaging characteristics

In total, 186 lesions from 179 female participants (mean age, 48 ± 11 years) with 115 malignant lesions and 71 benign lesions were enrolled after excluding 177 lesions (Fig. 1) from 17 tertiary centers and 13 provinces across China, allowing for the inclusion of a geographically diverse patient population. The mean age (SD) of the overall cohort was 49 ± 11 years, and all 179 of the patients were women (Table 1). The details of the overall pathologic distribution and of each cohort are presented in Additional file 1: Table S5. The frequencies of the PFB-CEUS and MP-MRI characteristics in the two data cohorts are described in Additional file 1: Table S6.

**Table 1:** Baseline characteristics of the study patients

Characteristic	Overall lesions (*n* = 186)	Development cohort (*n* = 151)	Validation cohort (*n* = 35)
Age (years)^b^	49 ± 11 (19, 76)	49 ± 11 (19, 76)	49 ± 10 (27, 68)
Age (years)^b^			
< 49	90 (48.4)	76 (50.3)	14 (40.0)
≧ 49	96 (51.6)	75 (49.7)	21 (60.0)
Body mass index (kg/m^2^)^b^	23.9 ± 3.5 (13.7, 37.0)	23.9 ± 3.3 (16.4, 37.0)	23.5 ± 4.2 (13.7, 34.1)
Body mass index (kg/m^2^)			
< 18.5	6 (3.4)	3 (2.1)	3 (9.1)
18.5 to < 25	110 (61.5)	91 (62.3)	19 (57.6)
≥ 25.0	63 (35.2)	52 (35.6)	11 (33.3)
Dense breast^e^			
Yes	137 (76.5)	107 (73.3)	30 (90.9)
No	42 (23.5)	39 (26.7)	3 (9.1)
Parity status			
Nulliparous	16 (8.9)	14 (9.6)	2 (6.1)
Parous	163 (91.1)	132 (90.4)	31 (93.9)
Menopausal status^c^			
Premenopausal	107 (59.8)	89 (61.0)	18 (54.5)
Postmenopausal	72 (40.2)	57 (39.0)	15 (45.5)
First-degree relatives with breast cancer^d^			
Presence	169 (94.4)	138 (94.5)	31 (93.9)
Absence	10 (5.6)	8 (5.5)	2 (6.1)
Diagnosis method			
Surgery	73 (39.2)	62 (41.1)	11 (31.4)
Core biopsy	113 (60.8)	89 (58.9)	24 (68.6)
Lesion size (cm)^a^	1.8 (1.2, 2.4)	1.8 (1.2, 2.5)	1.8 (1.3, 2.4)
Lesion size (cm)^a^			
< 1.5	65 (34.9)	52 (34.4)	13 (37.1)
≧ 1.5	121 (65.1)	99 (65.6)	22 (62.9)
Histologic type			
Benign	71 (38.1)	58 (38.4)	13 (37.1)
Malignant	115 (61.8)	93 (61.6)	22 (62.9)

Unless otherwise indicated, data are numbers of patients, and data in parentheses are percentages

^a^Data are medians, and data in parentheses are the interquartile range

^b^Data are means ± standard deviations, and data in parentheses are the range

^c^Menopausal status: Women aged 60 years or older, reporting a history of hysterectomy, or reporting no periods within the past 12 months without the use of hormonal contraceptives were categorized as postmenopausal. Women reporting regular periods (12–18 times in the last 12 months) without the use of hormonal contraceptives were categorized as premenopausal

^d^First-degree relatives (mother, sister, and daughter) with breast cancer

^e^Breast density assessed with Magnetic Resonance Imaging according to the ACR BI-RADS Magnetic Resonance Imaging categories a, b, c, d. In this study, c and d categories of breast density are defined as dense breast. On the other hand, the, a and b categories of breast density are defined as nondense breast

### Model development and performance

The univariate and multivariable logistic regression analyses of PFB-CEUS, MP-MRI, and the hybrid model are shown in Additional file 1: Tables S7, S8 and S9, respectively.

**Fig. 2:**
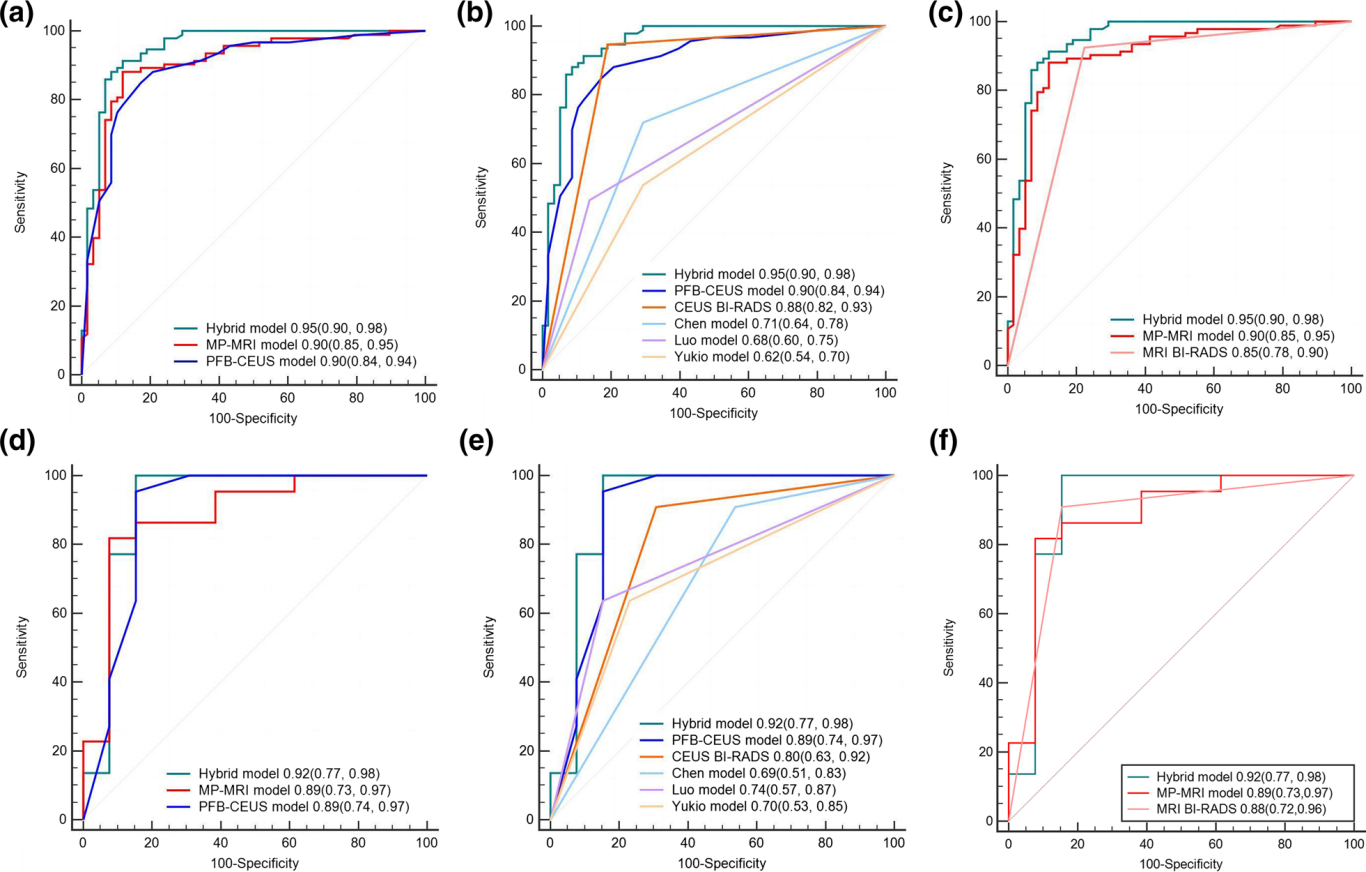
Receiver operating characteristic (ROC) curves of the models in the differentiation of breast lesions. **a**–**c** Results in the development cohort. **d**–**f** Results in the external validation cohort. *PFB-CEUS* perfluorobutane contrast-enhanced ultrasound, *MP-MRI* multiparametric magnetic resonance imaging

In the external validation model, the hybrid model showed an AUC similar to that of both MP-MRI and PFB-CEUS with an AUC of 0.92 ([95% CI 0.77, 0.98]). The PFB-CEUS model was shown to have an AUC of 0.89 ([95% CI 0.74,0.97]), which was comparable to the PFB-CEUS BI-RADS (AUC 0.80 [95% CI 0.63,0.92), *P* = 0.43; better than the Luo model (AUC 0.74 [95% CI 0.57,0.87], *P* = 0.01); higher than the Chen model (AUC 0.69 [95% CI 0.51, 0.83], *P* = 0.02); and superior to the Yukio model (AUC 0.70 [95% CI 0.53, 0.85], *P* = 0.01). In addition, the MP-MRI model was shown to have an AUC of 0.89 ([95% CI 0.73, 0.97]), which was competitive with ACR BI-RADS models (AUC 0.73 [95% CI 0.55, 0.87], *P* = 0.15). (Additional file 1: Table S10, Fig. 2).

**Fig. 3:**
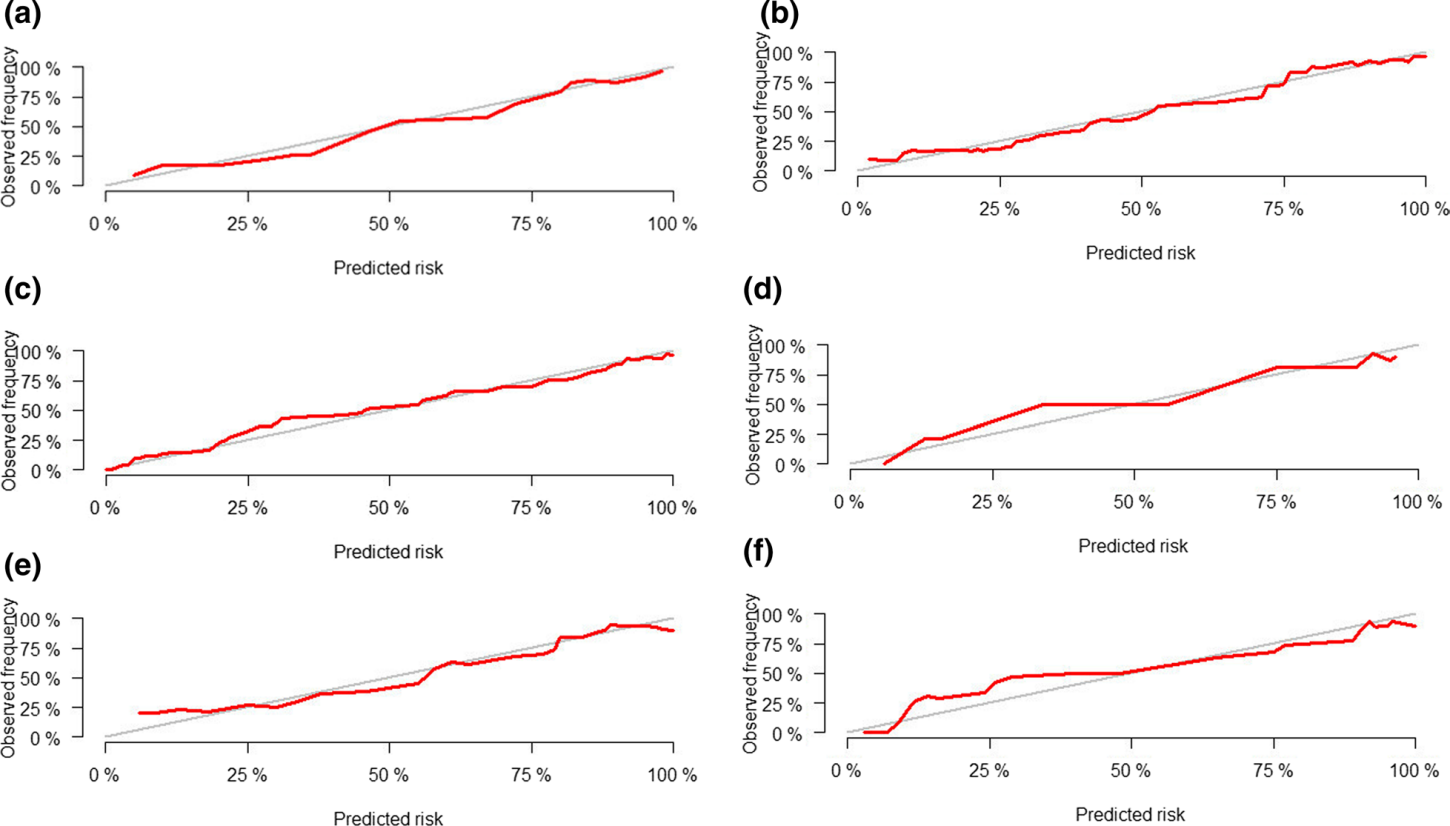
. Plot shows calibration of the PFB-CEUS, MP-MRI and hybrid models. **a** PFB-CEUS model result in the development cohort; **b** MP-MRI model result in the development cohort; **c** hybrid model result in the development cohort; **d** PFB-CEUS model result in the external validation cohort. **e** MP-MRI model result in the external validation cohort. **f** hybrid model result in the external validation cohort. *PFB-CEUS* perfluorobutane contrast-enhanced ultrasound, *MP-MRI* multiparametric magnetic resonance imaging

**Fig. 4:**
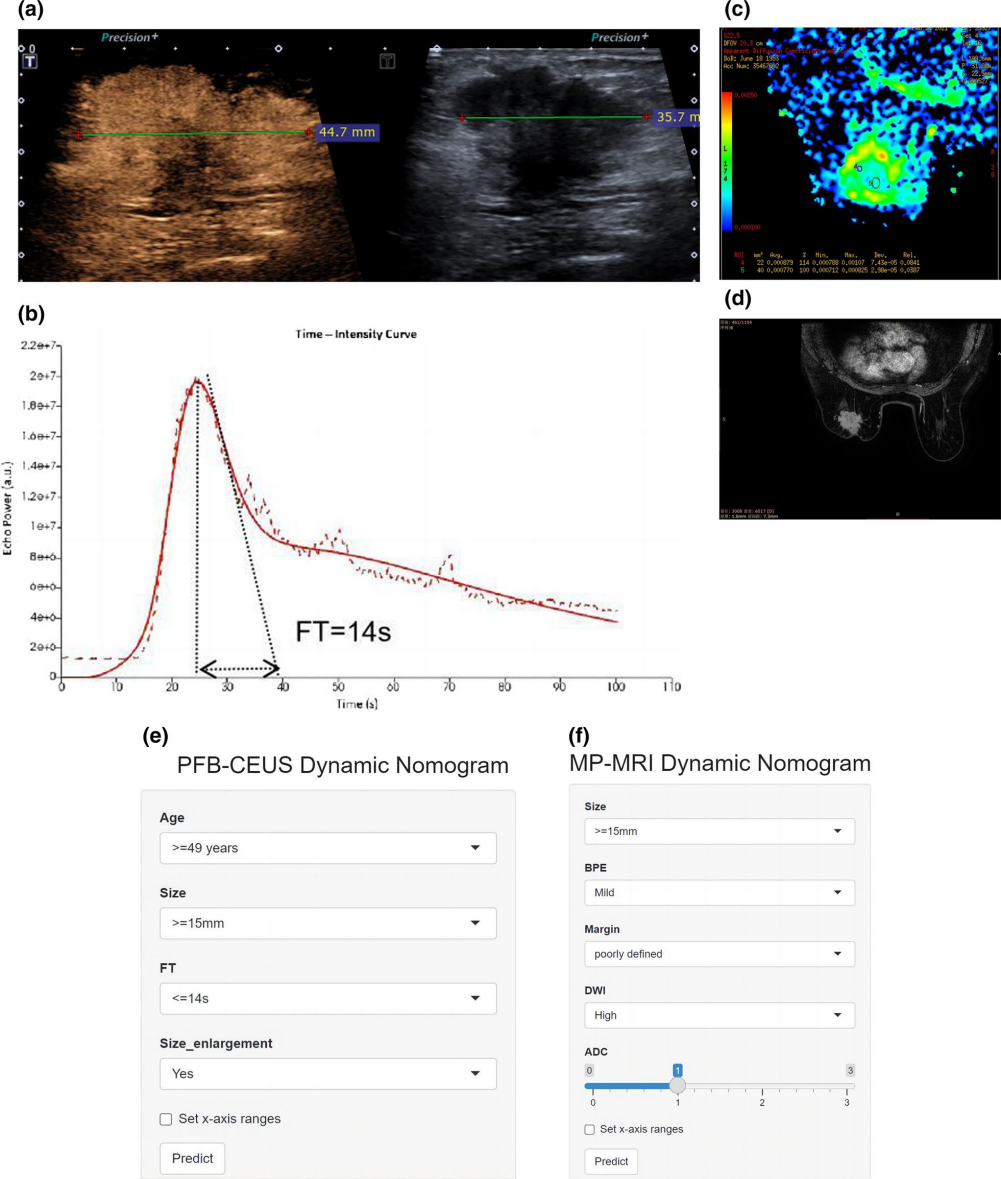
PFB-CEUS and MP-MRI images for breast cancer diagnosis. This is a 67-year-old woman with an invasive carcinoma by pathological diagnosis. **a** At 23 s, the PFB-CEUS had arrived at the peak intensity. The lesion showed a size enlargement on PFB-CEUS compared with gray ultrasound. **b** TIC of PFB-CEUS shows a 14 s fall time of the lesion. **c** DWI shows a high signal lesion with an ADC value of 0.77 * 10^–3^ mm^2^/s. **d** DCE-MRI shows patients with mild BPE and a lesion with a noncircumscribed margin. **e** PFB-CEUS Nomogram shows that this case had a 98% MP at the https://ceus.shinyapps.io/PFB-CEUS/link. **f** MP-MRI Nomogram shows that this case had a 95% MP at the https://ceus.shinyapps.io/MP-MRI/link. *MP-MRI* multiparametric magnetic resonance imaging, *PFB-CEUS* perfluorobutane contrast-enhanced ultrasound, *DWI* diffusion weighted imaging, *ADC* apparent diffusion coefficient, *DCE-MRI* dynamic contrast-enhanced magnetic resonance imaging, *BPE* background parenchymal enhancement, *TIC* time–intensity curve, *FT* fall time, *MP* malignant probability

**Fig. 5:**
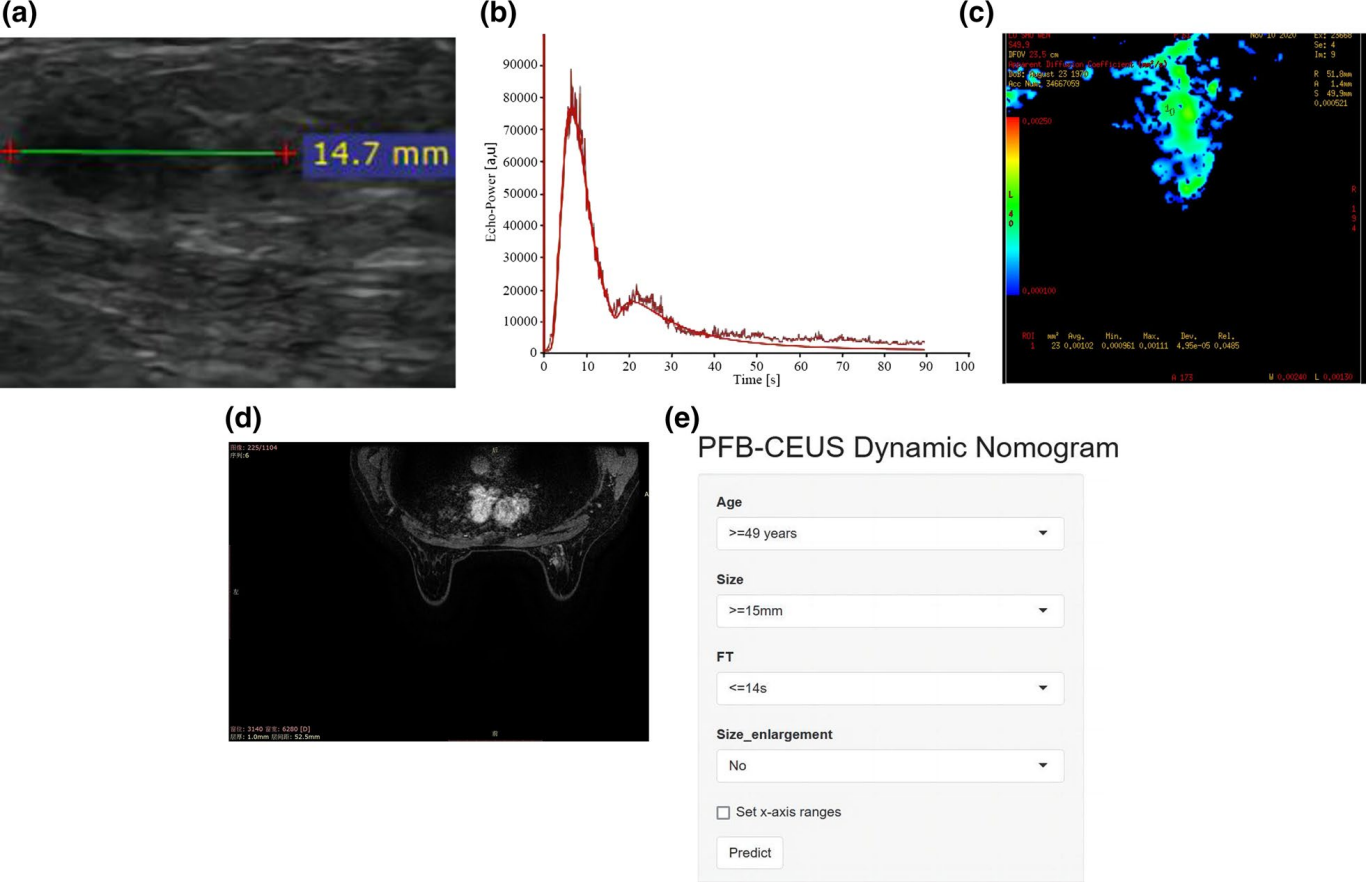
A breast cancer was diagnosed by PFB-CEUS as a malignant lesion but was diagnosed as a benign lesion by MP-MRI. This is a 50-year-old woman with an invasive carcinoma by pathological diagnosis. **a** At the 8th second, PFB-CEUS had arrived at the peak intensity. The lesion on PFB-CEUS was the same size as that on gray ultrasound. **b** TIC of PFB-CEUS shows a 6.6 s fall time of the lesion. **c** DWI shows a high signal lesion with an ADC value of 1.6 * 10^–3^ mm^2^/s. **d** DCE-MRI shows the patients with a marked BPE and a lesion with a noncircumscribed margin. **e** PFB-CEUS Nomogram shows that this case had an 82% MP at the https://ceus.shinyapps.io/PFB-CEUS/link. **f** MP-MRI Nomogram shows that this case had a 38% MP at the https://ceus.shinyapps.io/MP-MRI/link. *MP-MRI* multiparametric magnetic resonance imaging, *PFB-CEUS* perfluorobutane contrast-enhanced ultrasound, *DWI* diffusion weighted imaging, *ADC* apparent diffusion coefficient, *DCE-MRI* dynamic contrast-enhanced magnetic resonance imaging, *BPE* background parenchymal enhancement, *TIC* time–intensity curve, *FT* fall time, *MP* malignant probability

**Fig. 6:**
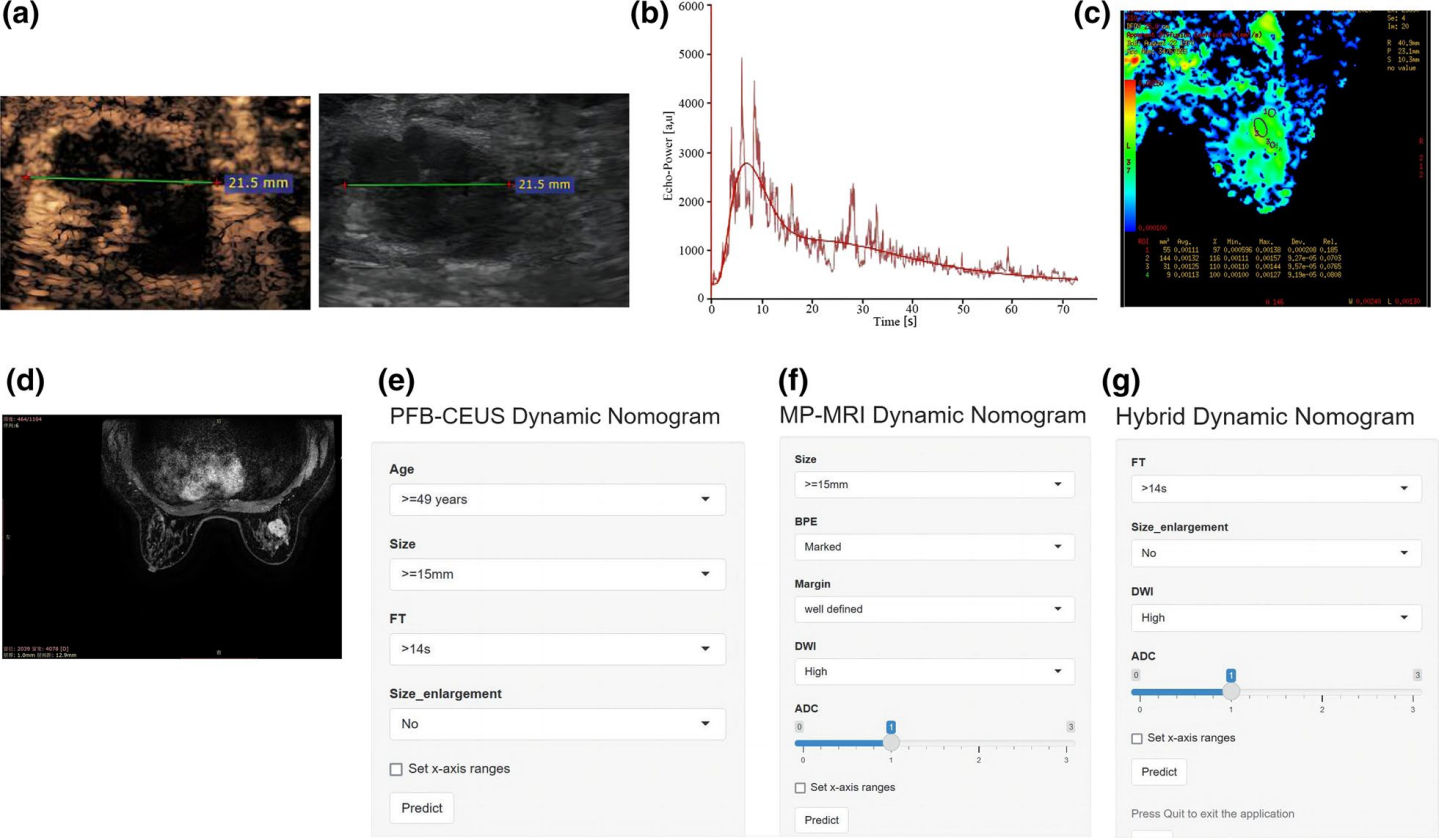
A benign breast lesion was diagnosed as a cancer by PFB-CEUS and MP-MRI but was diagnosed as a benign lesion by the hybrid model. This is a 50-year-old woman with ductal hyperplasia by pathological diagnosis. **a** At the 12th second, PFB-CEUS had arrived at the peak intensity. The lesion on PFB-CEUS was the same size as that on gray ultrasound. **b** TIC of PFB-CEUS shows a 14 s fall time of the lesion. **c** DWI shows a high signal lesion with an ADC value of 1.2 * 10^–3^ mm^2^/s. **d** DCE-MRI shows the patients with a marked BPE and a lesion with a circumscribed margin. **e** PFB-CEUS Nomogram shows that this case had a 36% MP at the https://ceus.shinyapps.io/PFB-CEUS/link. **f** MP-MRI Nomogram shows that this case had a 40% MP at the https://ceus.shinyapps.io/MP-MRI/link. **g** Hybrid Nomogram shows that this case had a 14% MP at the https://ceus.shinyapps.io/hybrid/link. *MP-MRI* multiparametric magnetic resonance imaging, *PFB-CEUS* perfluorobutane contrast-enhanced ultrasound, *DWI* diffusion weighted imaging, *ADC* apparent diffusion coefficient, *DCE-MRI* dynamic contrast-enhanced magnetic resonance imaging, *BPE* background parenchymal enhancement, *TIC* time–intensity curve, *FT* fall time, *MP* malignant probability

The PFB-CEUS, MP-MRI and hybrid models were well calibrated, and all showed statistical significance (*P* > 0.05) in the Hosmer‒Lemeshow test. The calibration plots are shown in Fig. 3. The decision curves are shown in Additional file 1: Figure S2. In addition, the respective dynamic nomograms of the PFB-CEUS, MP-MRI, and hybrid models are shown in the following links: https://ceus.shinyapps.io/PFB-CEUS/, https://ceus.shinyapps.io/MP-MRI/, https://ceus.shinyapps.io/Hybrid/. Figure 4 and Additional file 1: Figure S3 show a matched example in which the PFB-CEUS and MP-MRI nomograms showed a high malignant probability of breast cancer. Figure 5 and Additional file 1: Figure S4 show a matched example in which PFB-CEUS showed a high malignant probability of cancer, but MP-MRI nomograms showed a low malignant probability of cancer in which the lesion was diagnosed as invasive carcinoma by pathology. Figure 6 shows a matched example in which the PFB-CEUS and MP-MRI nomograms showed a high malignant probability of cancer, but the hybrid nomograms showed a low malignant probability of cancer in which the lesion was diagnosed as ductal hyperplasia by pathology.

### Comparison among the three models

#### PFB-CEUS versus MP-MRI model

The PFB-CEUS model showed a comparable discrimination ability for diagnosing breast cancer, with the MP-MRI model showing the same not only in the development cohort (AUC 0.90 [95% CI 0.84, 0.94]) versus (AUC 0.90 [95% CI: 0.85, 0.95], *P* = 0.80) but also in the validation cohort (AUC 0.89 [95% CI:0.74, 0.97]) versus (AUC 0.89 [95% CI: 0.73, 0.97], *P* = 0.85) (Additional file 1: Table S10).

#### PFB-CEUS versus hybrid model

The hybrid model showed a higher capacity to diagnose breast cancer compared with the PFB-CEUS model in the development cohort (AUC 0.95 [95% CI: 0.90, 0.98]) versus ((AUC 0.90, [95% CI 0.84,0.94]) *P* = 0.01) and a similar capacity with PFB-CEUS in the validation cohort ((AUC 0.92 [95% CI: 0.77, 0.98]) versus (AUC 0.89 [95% CI: 0.74, 0.97]), *P* = 0.29) (Additional file 1: Table S10).

#### MP-MRI versus hybrid model

The hybrid model demonstrated a competitive capacity for diagnosing breast cancer compared with the MP-MRI model ((AUC 0.95 [95% CI: 0.90, 0.98]) vs. (AUC 0.90 [95% CI: 0.85, 0.95]), respectively; *P* = 0.078), not only in the development cohort but also in the validation cohort ((AUC, 0.92 [95% CI: 0.77, 0.98]) vs. (AUC 0.89 [95% CI: 0.73,0.97]), respectively; *P* = 0.401) (Additional file 1: Table S10).

#### Model performance in subpopulations

Of all the subgroups dichotomized by age and menstrual status, the PFB-CEUS model showed a similar AUC to MP-MRI in the external validation cohort. No significant difference in AUC was noted between hybrid model comparisons of PFB-CEUS or MP-MRI in the external validation cohort (Additional file 1: Table S11). The diagnostic results for high-risk lesions (a lesion that would be appropriate for surgical consultation, including intraductal papilloma and phyllodes tumor) are presented in Additional file 1: Table S12.

### Model performance for diagnosing breast cancer compared with radiologists

BI-RADS 4A, 4B, and 4C were used as the cut points of malignant lesions for comparison.

#### PFB-CEUS

When compared with the on-site radiologists, the PFB-CEUS model achieved higher sensitivity in the BI-RADS 4C+ mode with lower specificity in the BI-RADS 4B+ mode. The hybrid model achieved higher sensitivity in the BI-RADS 4C+ mode with lower specificity in BI-RADS 4B+ and 4C+. When compared with the three senior reviewers, the PFB-CEUS model achieved higher sensitivity in the BI-RADS 4B+ and 4C+ modes, with lower specificity only in the BI-RADS 4C+ mode. The hybrid model achieved higher sensitivity in BI-RADS 4C+ mode and lower specificity in BI-RADS 4B+ mode (Table 2).

**Table 2:** Comparison of breast cancer diagnostic performance of each model with radiologists in different BI-RADS modes

	BI-RADS (3 vs. 4a+)				BI-RADS (3, 4a vs. 4b+)				BI-RADS (3, 4a, 4b vs. 4c+)			
	Se%	*P* value	Sp%	*P* value	Se%	*P* value	Sp%	*P* value	Se%	*P* value	Sp%	*P* value
PFB-CEUS												
On-site^a^	97.4 (112/115)		56.3 (40/71)		93.9 (108/115)		78.9 (56/71)		78.3 (90/115)		91.5 (65/71)	
	(92.6, 99.5)		(44.0, 68.1)		(87.9, 97.5)		(67.6, 87.7)		(69.6, 85.4)		(82.5, 96.8)	
Reviewers^b^	94.8 (109/115)		36.6 (26/71)		86.1 (99/115)		70.4 (50/71)		78.3 (90/115)		78.9 (56/71)	
	(89.0, 98.1)		(25.5, 48.9)		(78.4, 91.8)		(58.4, 80.7)		(69.6, 85.4)		(67.6, 87.7)	
CEUS model	NA	–^c^	NA	–^c^	97.4 (112/115)	0.344^c^	36.6 (26/71)	< 0.001^c^	88.7 (102/115)	0.029^c^	80.3 (57/71)	0.096^c^
		–^d^		–^d^	(92.6, 99.5)	0.001^d^	(25.5, 48.9)	< 0.001^d^	(81.4, 93.8)	0.023^d^	(69.1, 88.8)	–^d^
Hybrid model	100.0 (115/115)	–^c^	25.4 (18/71)	< 0.001^c^	100.0 (115/115)	–^c^	50.7 (36/71)	0.001^c^	90.4 (104/115)	0.004^c^	84.5 (60/71)	0.332^c^
	(96.8, 100.0)	–^d^	(15.8, 37.1)	0.229^d^	(96.8,100.0)	–^d^	(38.6, 62.8)	0.009^d^	(83.5, 95.1)	0.004^d^	(74.0, 92.0)	0.454^d^
MP-MRI												
On-site^a^	98.3 (113/115)		49.3 (35/71)		92.2 (106/115)		73.2 (52/71)		80.0 (92/115)		87.3 (62/71)	
	(93.9, 99.8)		(37.2, 61.4)		(85.7, 96.4)		(61.4, 83.1)		(71.5, 86.9)		(77.3, 94.0)	
Reviewers^b^	93.9 (108/115)		25.4 (18/71)		89.6 (103/115)		46.5 (33/71)		81.7 (94/115)		67.6 (48/71)	
	(87.9, 97.5)		(15.8, 37.1)		(82.5, 94.5)		(34.5, 58.7)		(73.5, 88.3)		(55.5, 78.2)	
MRI model	100.0 (115/115)	–^e^	11.3 (8/71)	< 0.001^e^	98.3 (113/115)	0.016^e^	40.8 (29/71)	< 0.001^e^	88.7 (102/115)	0.041^e^	80.3 (57/71)	0.332^e^
	(96.8, 100.0)	–^f^	(5.0, 21.0)	0.041^f^	(93.9, 99.8)	0.006^f^	(29.3,53.2)	0.627^f^	(81.4, 93.8)	0.134^f^	(69.1, 88.8)	0.078^f^
Hybrid model	100.0 (115/115)	–^e^	25.4 (18/71)	0.002^e^	100.0 (115/115)	–^e^	50.7 (36/71)	0.004^e^	90.4 (104/115)	0.017^e^	84.5 (60/71)	0.815^e^
	(96.8, 100.0)	–^f^	(15.8, 37.1)	–^f^	(96.8, 100.0)	–^f^	(38.6, 62.8)	0.711^f^	(83.5, 95.1)	0.052^f^	(74.0, 92.0)	0.023^f^

The model’s sensitivity was determined by setting the model’s specificity to match the on-site/reviewers’ specificity; the model’s specificity was determined by setting the model’s sensitivity to match the on-site/reviewers’ sensitivity. *Se* sensitivity, *Sp* specificity. NA means the PFB-CEUS model’s breast lesion diagnosis as BI-RADS 3 grade is absent. Data in parentheses are the numerator and denominator and the 95% confidence intervals

^a^25 radiologists who performed PFB-CEUS and 27 radiologists who performed MP-MRI

^b^3 radiologists who reviewed the stored PFB-CEUS and 3 radiologists who reviewed the stored MP-MRI images

^c^*P* value for comparisons with the diagnostic results of PFB-CEUS radiologists on-site

^d^*P* value for comparisons with the diagnostic results of the PFB-CEUS reviewers

^e^*P* value for comparisons with the diagnostic results of MP-MRI radiologists on-site

^f^*P* value for comparisons with diagnostic results of MP-MRI reviewers

#### MP-MRI

When compared with the on-site radiologists, the MP-MRI model achieved higher sensitivity in all three modes with lower specificity in the BI-RADS 4A+ and 4B+ modes, as well as the hybrid model. When compared with the three senior reviewers, the MP-MRI model achieved higher sensitivity in BI-RADS 4B+ mode and lower specificity in BI-RADS 4A+ mode. The hybrid model achieved comparable sensitivity in the BI-RADS 4C+ mode and higher specificity in the BI-RADS 4C+ mode (Table 2).

### Model performance with false-positive and false-negative correction rate

#### PFB-CEUS

**Fig. 7:**
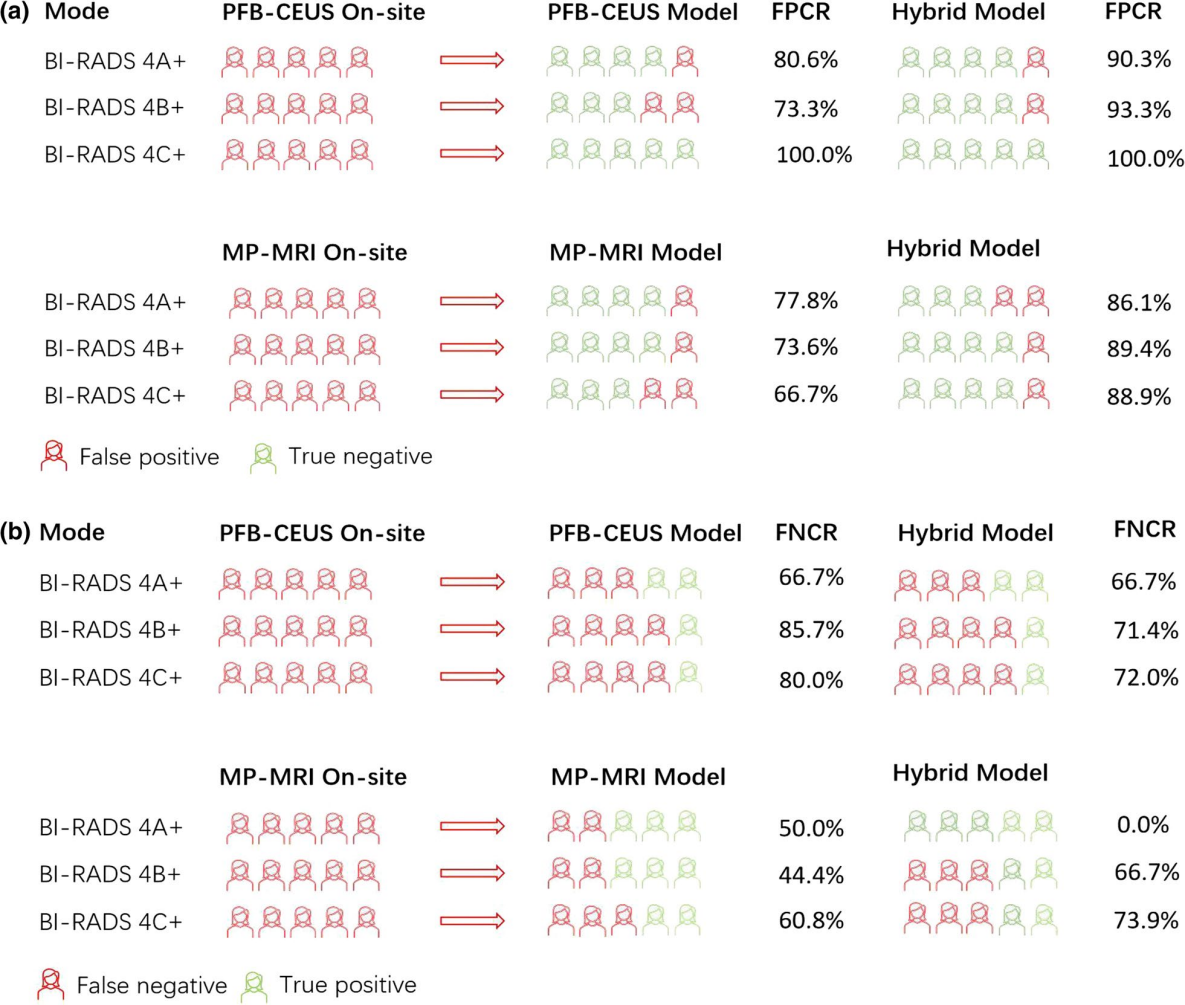
FPCR and FNCR for on-site radiologists with models. **a** FPCR for on-site radiologists with models. **b** FNCR for on-site radiologists with models. On-site presented the radiologists who performed PFB-CEUS and MP-MRI. *FPCR* false-positive correction rate, *FNIR* false-negative correction rate

The false-positive correction rates of the PFB-CEUS and hybrid models were 80.6% and 90.3% for the on-site results and 82.2% and 88.9% for the reviewers’ results based on the BI-RADS 4A+ modes, respectively. The false-negative correction rates of the PFB-CEUS and hybrid models were 66.7% and 66.7% for the on-site results and 83.3% for the reviewers’ results in the BI-RADS 4A+ mode, respectively (Additional file 1: Table S13) (Fig. 7).

#### MP-MRI

The false-positive correction rates of the MP-MRI and hybrid model were 77.7% and 86.1% for the on-site results and 83.0% and 90.5% for the reviewers’ results in the BI-RADS 4A+ mode, respectively. The false-negative correction rates of the MP-MRI and hybrid models were 50.0% and 0.0% for the on-site results and 57.1% and 42.8% for the reviewers’ results in the BI-RADS 4A+ mode, respectively (Additional file 1: Table S13) (Fig. 7).

## Discussion

MP-MRI has better sensitivity and specificity for the detection of breast cancer than dynamic contrast-enhanced MRI (DCE-MRI) [29]. However, this technology was not always available and was associated with prohibitively high costs for use as a routine diagnostic tool [30]. There is an increasing demand to develop a surrogate, easy-to-implement method to diagnose breast lesions. Our study is the first to compare the diagnostic performance between PFB-CEUS and MP-MRI, both advanced breast cancer diagnosis modalities, for the diagnosis of breast cancer.

In this study, the AUC for the PFB-CEUS model was similar to that of the MP-MRI model, as well as in the subgroup analysis The hybrid model could improve the sensitivity and specificity of PFB-CEUS on-site radiologists in all three modes, as well as MP-MRI. The FPCR and FNCR were excellent for PFB-CEUS, MP-MRI, and the hybrid model. The results indicate that PFB-CEUS could potentially improve the diagnostic ability of MP-MRI for breast cancer, and when patients are restricted by the contraindications of MP-MRI, PFB-CEUS could be performed as an alternative examination method for patients with breast lesions.

Over the past decade, several comparative studies to investigate the ability of SHF-CEUS and DCE-MRI for breast cancer diagnosis have been published, and these have demonstrated inconsistent results. (Additional file 1: Table S1). Half indicated that SHF-CEUS showed higher sensitivity and specificity than DCE-MRI, and the remainder presented contrasting results. In all these studies, there was no rigorous imaging feature review or selection and a lack of evaluation of interobserver variability between radiologists and inclusion analysis of quantitative SHF-CEUS indicators. Compared with the previous retrospective cohort study between SHF-CEUS and DCE-MRI for breast cancer, it is worth mentioning that PFB-CEUS and MP-MRI images were prospectively collected according to the standard protocol in our study.

Notably, our study has confirmed the theoretical advantages of PFB, which has a high resonance frequency and stable outer shell, leading to the depiction of clear enhancement signals such as tumor size and duration and improving the CEUS image quality in linear transducers. Enhancement size enlargement and FT became stable indicators through a bootstrap analysis that excluded the zero value, ensuring a more accurate estimation of PFB-CEUS. Compared with the previously constructed CEUS model, our study showed a higher AUC than all the others in both the development cohort and validation cohorts. In addition, MP-MRI was set as the control with PFB-CEUS in the present study, which can reflect the diffusion of water molecules in the intracellular and extracellular spaces and can lead to a higher specificity than DCE-MRI in the diagnosis of malignant lesions due to the high cell density and the small extracellular space in malignant lesions [29, 31, 32]. Encouragingly, PFB-CEUS did not sacrifice the diagnostic performance for breast cancer compared to MP-MRI with PFB-CEUS superior contrast resolution.

When PFB was used as an agent to compare with MRI for breast cancer, only one result was reported, namely that PFB-CEUS reached a similar AUC compared with DCE-MRI in 127 patients [14]. Our study involved patients who were prospectively enrolled from 17 tertiary centers across China, providing for a head-to-head comparative study with a relatively large sample size that guaranteed the generalization results. Rigorous validation, including the 1000-sample bootstrap, five- and tenfold internal validation and independent external validation, ensured robust and reproducible results, even when using different device settings, and the comprehensive features were analyzed for the ability to distinguish breast cancer by using clinical parameters and qualitative and quantitative analysis of perinodular and intranodular features selected from PFB-CEUS and MP-MRI.

Compared with the radiologists, the implementation of all three models was feasible in daily practice, requiring less time and effort to collect and analyze clinical and imaging characteristics (only 3–5 variables). Freely available online nomograms with relatively reliable results are structured for simple daily practice and are accessible to three different websites.

Furthermore, a false-positive imaging diagnosis may lead to unnecessary biopsy invasion, patient anxiety, and health care costs. BI-RADS 4A is usually recommended for use in the clinic as the cutoff point for biopsy. In our study, the FPCR of PFB-CEUS, MP-MRI, and the hybrid model was excellent, particularly when BI-RADS 4A was used as the cutoff point to diagnose positive lesions, indicating that the models’ FPCR could supply a reference to appropriately avoid overdiagnosis for probable benign lesions.

Our study had several limitations. First, the absolute accuracy and stability of the models may have been influenced by the relatively small sample size. The subgroup analysis in the different lesion size and breast density groups failed to achieve statistical results because of the small sample size in some groups, although this was calculated in the design of this prospective study. Second, because we enrolled only lesions with BI-RADS grades 3, 4, and 5 in the grayscale US, the prevalence of breast cancer in these participants may be higher than that found during the usual diagnostic examination for all surveillance-positive lesions. Therefore, the diagnostic performance of PFB-CEUS and MP-MRI is potentially biased. Last, the patients enrolled in this study were only Chinese without populations from other countries. In the future, a worldwide range trial could be conducted.

In conclusion, the PFB-CEUS model was not only more efficient but also showed similar diagnostic ability as the MP-MRI model. Using PFB-CEUS and MP-MRI as joint diagnostics could further strengthen the ability to better characterize breast cancer. This suggests that not only can PFB-CEUS be used as a surrogate method to diagnose breast lesions but also that when these models are used as an aid to radiologists in clinical practice, they can save time and effort and avoid overdiagnosis.

## Supplementary Information

**Additional file 1.** Supplementary tables and figures.

## Data Availability

Data generated or analyzed during the study are available from the corresponding author by request.
