# Supplementary material for: Head-to-head comparison of perfluorobutane contrast-enhanced US and multiparametric MRI for breast cancer: a prospective, multicenter study
Source: Breast Cancer Res. 2023 May 30;25:61. doi: 10.1186/s13058-023-01650-3 (PMC10228005; doi:10.1186/s13058-023-01650-3)
Supplement: Supplementary file 1 — Additional file 1. Supplementary tables and figures. [file 13058_2023_1650_MOESM1_ESM.docx]

**TABLE S1.** Summary of published studies comparing the diagnostic performance between PFB-CEUS and MP-MRI

| Author | Year | Numbers of lesions | Centers | Design | UCA | MRI method | Index | CEUS (%) | MRI (%) | *P* Value* |
| --- | --- | --- | --- | --- | --- | --- | --- | --- | --- | --- |
| Du, J et al. (1) | 2012 | 61 | Single | Retrospective | Sulfur hexafluoride | DCE-MRI | Se | 75.8 | 97.0 | <.05 |
|  |  |  |  |  |  |  | Sp | 82.1 | 85.7 | >.05 |
| Miyamoto Y et al. (2) | 2014 | 127 | Multi | Prospective | Perfluorobutane | DCE-MRI | Se | 91.4 | 84.8 | .15 |
|  |  |  |  |  |  |  | Sp | 85.4 | 63.0 | <.001 |
| Pan J et al. (3) | 2020 | 52 | Single | Retrospective | Sulfur hexafluoride | DCE-MRI | Se | 95.0 | 90.0 | >.05 |
|  |  |  |  |  |  |  | Sp | 96.8 | 78.1 | .031 |
| Li, C et al. (4) | 2020 | 406 | Single | Retrospective | Sulfur hexafluoride | DCE-MRI | Se | 98.1 | 98.6 | 1.000 |
|  |  |  |  |  |  |  | Sp | 62.9 | 80.4 | <.001 |
| Liu, W et al. (5) | 2020 | 252 | Single | Retrospective | Sulfur hexafluoride | DCE-MRI | Se | 94.2 | 92.3 | >.05 |
|  |  |  |  |  |  |  | Sp | 81.8 | 89.2 | >.05 |

Note.—UCA = ultrasound contrast agent, Se = sensitivity, Sp = specificity, DCE-MRI =Dynamic contrast enhanced- Magnetic Resonance Imaging

^*^ *P* value for comparisons between CEUS and MRI.

**TABLE S2.** Characteristics of centers in the development and validation cohorts

| Groups | No. | Center | City | Type |
| --- | --- | --- | --- | --- |
| Development cohort | 1 | Chinese PLA General Hospital | Beijing | Academic |
|  | 2 | Zhongda Hospital Southeast University | Nanjing | Academic |
|  | 3 | The Affiliated Hospital of Putian University | Putian | Academic |
|  | 4 | Huashan Hospital of Fudan University | Shanghai | Academic |
|  | 5 | Guangxi Medical University Affiliated Tumor Hospital | Nanning | Academic |
|  | 6 | Beijing Friendship Hospital, Capital Medical University | Beijing | Academic |
|  | 7 | The Second Affiliated Hospital of Harbin Medical University | Harbin | Academic |
|  | 8 | China-Japan Union Hospital of Jilin University | Changchun | Academic |
|  | 9 | The First Affiliated Hospital of Harbin Medical University | Harbin | Academic |
|  | 10 | Jiangsu Province Hospital of Chinese Medicine | Nanjing | Academic |
|  | 11 | Shengjing Hospital of Chinese Medical University | Shenyang | Academic |
|  | 12 | The Affiliated Hospital of Inner Mongolia Medical University | Hohhot | Academic |
|  | 13 | The First Affiliated Hospital of Xinxiang Medical University | Xinxiang | Academic |
| Validation cohort | 1 | The Fourth Hospital of Hebei Medical University | Shijiazhuang | Academic |
|  | 2 | Third Xiangya Hospital of Central South University | Changsha | Academic |
|  | 3 | Peking University Third Hospital | Beijing | Academic |
|  | 4 | Zhengzhou Central Hospital | Zhengzhou | Academic |

**TABLE S3.** PFB-CEUS devices and probes

| Ultrasound devices | Model | Country | Probe |
| --- | --- | --- | --- |
| Conon | Aplio 500 | Japan | 14L5 |
| Conon | Aplio i800 | Japan | i18LX5 |
| Hitachi | Aloka | Japan | L441 |
| GE | LOGIQ E9 | American | ML 6-15 |
| GE | LOGIQ E9 | American | 9 L |
| GE | LOGIQ E20 | American | 9 L |
| Mindray | Resona7 | China | L9-3U |
| SAMSUNG | RS80A | Korea | LA2-9A |
| SAMSUNG | RS85 | Korea | LA2-9A |
| Supersonic | Aixplorer | French | SL10-2 |
| Supersonic | Aixplorer V | French | SL10-2 |
| Philips | IU-22 | American | L12-5 |
| Philips | IE33 | American | L12-5 |
| Philips | EPIQ7 | American | eL18-4 |
| Siemens | Sequaia | Germany | 9 L |

**TABLE S4.** Multiparameter MRI devices

| MRI devices | Model | Field strength | Country |
| --- | --- | --- | --- |
| GE | Single Excite | 1.5T | American |
| GE | 750 W | 3.0T | American |
| Siemens | Prisma | 3.0T | Germany |
| Siemens | VIDA | 3.0T | Germany |
| Siemens | Skyra | 3.0T | Germany |
| Siemens | Spectra | 3.0T | Germany |
| PHILIPS | Ingenia | 3.0T | Netherlands |
| PHILIPS | Ingenia CX | 3.0T | Netherlands |

**TABLE S5.** Numbers and histologic types of breast lesions

| Histologic type: No. (%) | Overall (n=186) | Development cohort (n=151) | Validation cohort (n=35) |
| --- | --- | --- | --- |
| Benign | 71 (38.2) | 58 (38.4) | 13 (37.1) |
| Fibroadenoma | 27 (14.5) | 23 (39.6) | 4 (30.7) |
| Adenosis | 15 (8.0) | 13 (22.4) | 2 (15.3)) |
| Intraductal papilloma | 9 (4.8) | 4 (6.8) | 5 (38.4) |
| Hyperplasia | 9 (4.8) | 8 (13.7) | 1 (7.6) |
| Adenosis with fibroadenoma | 5 (2.7) | 5 (8.6) | 0 (0.0) |
| Chronic mastitis | 2 (1.1) | 2 (3.4) | 0 (0.0) |
| Plasma-cell mastitis | 1 (0.5) | 1 (1.7) | 0 (0.0) |
| Cyst | 1 (0.5) | 1 (1.7) | 0 (0.0) |
| Granulomatous mastitis | 1 (0.5) | 0 (0.0) | 1 (7.6) |
| Phyllodes tumor | 1 (0.5) | 1 (1.7) | 0 (0.0) |
| Malignant | 115 (61.8) | 93 (61.5) | 22 (62.8) |
| Invasive breast cancers | 107 (57.5) | 86 (57.0) | 21 (60.0) |
| Ductal carcinoma in situ | 8 (4.3) | 7 (44.6) | 1 (22.9) |

**TABLE S6.** Imaging findings of breast lesions

| Variable: No. (%) | PFB-CEUS | | | MP-MRI | | |
| --- | --- | --- | --- | --- | --- | --- |
|  | Overall (n=186) | Development cohort (n=151) | Validation cohort (n=35) | Overall (n=186) | Development cohort (n=151) | Validation cohort (n=35) |
| **Intranodular features** |  |  |  |  |  |  |
| Margin |  |  |  |  |  |  |
| Circumscribed | 69 (37.1) | 56 (37.1) | 13 (37.1) | 71 (38.2) | 57 (37.7) | 14 (40.0) |
| Non-circumscribed | 117 (62.9) | 95 (62.9) | 22 (62.9) | 115 (61.8) | 94 (62.3) | 21 (60.0) |
| Shape |  |  |  |  |  |  |
| Round/Oval | 125 (67.2) | 99 (65.6) | 26 (74.3) | 56 (30.1) | 46 (30.5) | 10 (28.6) |
| Irregular | 61 (32.8) | 52 (34.4) | 9 (25.7) | 130 (69.9) | 105 (69.5) | 25 (71.4) |
| Uptake pattern |  |  |  |  |  |  |
| Nonenhancement | 4 (2.2) | 3 (2.0) | 1 (2.9) | 8 (4.3) | 3 (2.0) | 5 (14.3) |
| Centripetal | 84 (45.2) | 66 (43.7) | 18 (51.4) | 75 (40.3) | 55 (36.4) | 20 (57.1) |
| Centrifugal | 5 (2.7) | 3 (2.0) | 2 (5.7) | 27 (14.5) | 25 (16.6) | 2 (5.7) |
| Diffuse | 93 (50.0) | 79 (52.3) | 14 (40.0) | 76 (40.9) | 68 (45.0) | 8 (22.9) |
| Homogeneous pattern |  |  |  |  |  |  |
| Presence | 60 (32.3) | 48 (31.8) | 12 (34.3) | 54 (29.0) | 43 (28.5) | 11 (31.4) |
| Absence | 126 (67.7) | 103 (68.2) | 23 (65.7) | 132 (71.0) | 108 (71.5) | 24 (68.6) |
| Rim-like pattern |  |  |  |  |  |  |
| Absence | 169 (90.9) | 137 (90.7) | 32(91.4) | 143(76.9) | 116(76.8) | 27(77.1) |
| Presence | 17(9.1) | 14(9.3) | 3(8.6) | 43(23.1) | 35(23.2) | 8(22.9) |
| Claw-shaped pattern |  |  |  |  |  |  |
| Absence | 145 (78.0) | 120 (79.5) | 25 (71.4) | 126 (67.7) | 101 (66.9) | 25 (71.4) |
| Presence | 41 (22.0) | 31 (20.5) | 10 (28.6) | 60 (32.3) | 50 (33.1) | 10 (28.6) |
| Type of lesion |  |  |  |  |  |  |
| Focus/foci only | - | - | - | 4 (2.2) | 3 (2.0) | 1 (2.9) |
| Mass | - | - | - | 143 (76.9) | 122 (80.8) | 21 (60.0) |
| Nonmass enhancement | - | - | - | 34 (18.3) | 2 4(15.9) | 10 (28.6) |
| No enhancement | - | - | - | 5 (2.7) | 2 (1.3) | 3 (8.6) |
| Kinetics—initial phase |  |  |  |  |  |  |
| Slow | - | - | - | 37 (19.9) | 26 (17.2) | 11 (31.4) |
| Medium | - | - | - | 72 (38.7) | 66 (43.7) | 6 (17.1) |
| Rapid | - | - | - | 77 (41.4) | 59 (39.1) | 18 (51.4) |
| Kinetics—delayed phase |  |  |  |  |  |  |
| Persistent | - | - | - | 47 (25.3) | 35 (23.2) | 12 (34.3) |
| Plateau | - | - | - | 63 (33.9) | 57 (37.7) | 6 (17.1) |
| Washout | - | - | - | 76 (40.9) | 59 (39.1) | 17 (48.6) |
| T1W |  |  |  |  |  |  |
| High | - | - | - | 15 (8.1) | 1 1(7.3) | 4 (11.4) |
| Equal | - | - | - | 76 (40.9) | 60 (39.7) | 16 (45.7) |
| Low | - | - | - | 95 (51.1) | 80 (53.0) | 15 (42.9) |
| T2W |  |  |  |  |  |  |
| Low | - | - | - | 35 (18.8) | 33 (21.9) | 2 (5.7) |
| High | - | - | - | 120 (64.5) | 93 (61.6) | 27 (77.1) |
| Equal | - | - | - | 31 (16.7) | 25 (16.6) | 6 (17.1) |
| DWI |  |  |  |  |  |  |
| Other | - | - | - | 30 (16.1) | 29 (19.2) | 1 (2.9) |
| High | - | - | - | 156 (83.9) | 12 2(80.8) | 34 (97.1) |
| ADC value (10−3 mm2/s) | - | - | - | 1.1 (0.8,1.5) | 1.1 (0.9,1.5) | 1.0 (0.7,1.4) |
| AT > 12.0 s |  |  |  |  |  |  |
| Absence | 97 (52.2) | 78 (51.7) | 19 (54.3) | - | - | - |
| Presence | 89 (47.8) | 73 (48.3) | 16 (45.7) | - | - | - |
| RT≦8.1 s |  |  |  |  |  |  |
| Absence | 88 (47.3) | 73 (48.3) | 15 (42.9) | - | - | - |
| Presence | 98 (52.7) | 78(51.7) | 20 (57.1) | - | - | - |
| TTP≦21.8 s |  |  |  |  |  |  |
| Absence | 88 (47.3) | 71 (47.0) | 17 (48.6) | - | - | - |
| Presence | 98 (52.7) | 80 (53.0) | 18 (51.4) | - | - | - |
| mTT≦15.8 s |  |  |  |  |  |  |
| Absence | 66 (35.5) | 52 (34.4) | 14 (40.0) | - | - | - |
| Presence | 120 (64.5) | 99 (65.6) | 21 (60.0) | - | - | - |
| FT≦14.1 s |  |  |  |  |  |  |
| Absence | 71 (38.2) | 56 (37.1) | 15 (42.9) | - | - | - |
| Presence | 115 (61.8) | 95 (62.9) | 20 (57.1) | - | - | - |
| Derivation of washin time^&^ |  |  |  |  |  |  |
| Synchronous | 22 (11.8) | 15 (9.9) | 7 (20.0) | - | - | - |
| Earlier | 130 (69.9) | 104 (68.9) | 26 (74.3) | - | - | - |
| Later | 34 (18.3) | 32 (21.2) | 2 (5.7) | - | - | - |
| Entirely washout time of lesions > 5 mins |  |  |  |  |  |  |
| Absence | 86 (46.2) | 78 (51.7) | 8 (22.9) | - | - | - |
| Presence | 100 (53.8) | 73 (48.3) | 27 (77.1) | - | - | - |
| Enhancement degree |  |  |  |  |  |  |
| Nonenhancement | 4 (2.2) | 3 (2.0) | 1 (2.9) | - | - | - |
| Hyperenhancement | 132 (71.0) | 105 (69.5) | 27 (77.1) | - | - | - |
| Isoenhancement | 18 (9.7) | 14 (9.3) | 4 (11.4) | - | - | - |
| Hypoenhancement | 32 (17.2) | 29 (19.2) | 3 (8.6) | - | - | - |
| Perfusion defects |  |  |  |  |  |  |
| Absence | 88 (47.3) | 71 (47.0) | 17 (48.6) | - | - | - |
| Presence | 98 (52.7) | 80 (53.0) | 18 (51.4) | - | - | - |
| Size enlargement^*^ |  |  |  |  |  |  |
| Absence | 89 (47.8) | 75 (49.7) | 14 (40.0) | - | - | - |
| Presence | 97 (52.2) | 76 (50.3) | 21 (60.0) | - | - | - |
| **Perinodular features** |  |  |  |  |  |  |
| Nourishing vessels |  |  |  |  |  |  |
| Absence | 93 (50.0) | 83 (55.0) | 10 (28.6) | - | - | - |
| Presence | 93 (50.0) | 68 (45.0) | 25 (71.4) | - | - | - |
| BPE |  |  |  |  |  |  |
| Minimal | - | - | - | 53 (28.5) | 46 (30.5) | 7 (20.0) |
| Mild | - | - | - | 82 (44.1) | 63 (41.7) | 19 (54.3) |
| Moderate | - | - | - | 25 (13.4) | 23 (15.2) | 2 (5.7) |
| Marked | - | - | - | 26 (14.0) | 19 (12.6) | 7 (20.0) |
| Breast density^^^ |  |  |  |  |  |  |
| Yes |  |  |  | 137 (76.5) | 107 (73.3) | 30 (90.9) |
| No |  |  |  | 42 (23.5) | 39 (26.7) | 3 (9.1) |

Note.—Data are numbers of patients, and data in parentheses are percentages. BPE = Background Parenchymal Enhancement, DWI = Diffusion Weighted Imaging, ADC = Apparent Diffusion Coefficient, AT = Arrival Time, RT = Rise Time, TTP = Time To Peak, mTT = mean Transit Time, FT = fall time.

^&^ The wash-in time of breast lesions compared with that of the breast mammary gland.

^*^ The lesion size measured in the contrast-enhanced ultrasound is larger than that in the gray ultrasound.

^^^Breast density assessed with Magnetic Resonance Imaging according to the ACR BI-RADS Magnetic Resonance Imaging categories a, b, c, d. In this study, c and d categories of breast density isare defined as dense breastbreasts. On the other hand, the, a and b categories of breast density isare defined as non-dense breastnon-dense breasts.

**TABLE S7** Logistic analysis of PFB-CEUS model variables for breast lesions.

|  | Univariable | | | Multivariable | | |
| --- | --- | --- | --- | --- | --- | --- |
| Variable | Odds Radio | 95% CI | *P* Value | Odds Radio | 95% CI | *P* Value |
| Age (y) |  |  |  |  |  |  |
| <49 | Reference |  |  | Reference |  |  |
| ≧49 | 3.627 | (1.941, 6.780) | <.001 | 4.189 | (1.810, 9.697) | .001 |
| Menopausal status^‡^ |  |  |  |  |  |  |
| Before | Reference |  |  |  |  |  |
| After | 2.699 | 1.412, 5.519 | .003 |  |  |  |
| Nulliparity |  |  |  |  |  |  |
| No | Reference |  |  |  |  |  |
| Yes | 1.698 | 0.607, 4.749 | .313 |  |  |  |
| **Intranodular features** |  |  |  |  |  |  |
| Diameter(cm) |  |  |  |  |  |  |
| <1.5 | Reference |  |  | Reference |  |  |
| ≧1.5 | 3.381 | (1.799, 6.355) | <.001 | 3.381 | (1.285, 6.940) | .011 |
| Time |  |  |  |  |  |  |
| AT > 12.0 s |  |  |  |  |  |  |
| Absence | Reference |  |  |  |  |  |
| Presence | 2.517 | 1.276, 4.967 | .008 |  |  |  |
| RT≦8.1 s |  |  |  |  |  |  |
| Absence | Reference |  |  |  |  |  |
| Presence | 6.936 | 3.295, 14.602 | <.001 |  |  |  |
| TTP≦21.8 s |  |  |  |  |  |  |
| Absence | Reference | | |  |  |  |
| Presence | 3.913 | 1.952, 7.844 | <.001 |  |  |  |
| MTT≦15.8 s |  |  |  |  |  |  |
| Absence | Reference | | |  |  |  |
| Presence | 9.162 | 4.244, 19.777 | <.001 |  |  |  |
| FT≦14.1 s |  | | |  |  |  |
| Absence | Reference | | | Reference |  |  |
| Presence | 9.176 | 4.296, 19.617 | <.001 | 9.372 | 3.358, 26.152 | <.001 |
| Derivation of washin time^&^ |  |  |  |  |  |  |
| Synchronous | Reference | | |  |  |  |
| Earlier | 2.353 | 0.778, 7.026 | .125 |  |  |  |
| Later | 1.143 | 0.335, 3.904 | .831 |  |  |  |
| Entire washout time of lesions > 5 mins |  |  |  |  |  |  |
| Absence | Reference | | |  |  |  |
| Presence | 1.987 | 1.017, 3.881 | .044 |  |  |  |
| Internal enhancement characteristics |  |  |  |  |  |  |
| Enhancement degree |  |  |  |  |  |  |
| Nonenhancement | Reference |  |  |  |  |  |
| Hyperenhancement | 1.091 | 0.096, 12.461 | .944 |  |  |  |
| Isoenhancement | 0.5 | 0.036, 6.862 | .604 |  |  |  |
| Hypoenhancement | 0.353 | 0.029, 4.350 | .416 |  |  |  |
| Uptake pattern |  |  |  |  |  |  |
| Nonenhancement | Reference | | |  |  |  |
| Centripetal | 0.769 | 0.066, 8.920 | .834 |  |  |  |
| Centrifugal | 1 | 0.034, 29.807 | 1 |  |  |  |
| Diffuse | 0.817 | 0.071, 9.399 | .871 |  |  |  |
| Homogeneous pattern |  |  |  |  |  |  |
| Presence | Reference | | |  |  |  |
| Absence | 1.575 | 0.784, 3.163 | .202 |  |  |  |
| Rim-like pattern |  |  |  |  |  |  |
| Absence | Reference |  |  |  |  |  |
| Presence | 0.431 | 0.141, 1.313 | .139 |  |  |  |
| Claw-shaped pattern |  |  |  |  |  |  |
| Absence | Reference |  |  |  |  |  |
| Presence | 12.687 | 2.897, 55.574 | .001 |  |  |  |
| Perfusion defects |  |  |  |  |  |  |
| Absence | Reference |  |  |  |  |  |
| Presence | 1.911 | 0.983, 3.712 | .056 |  |  |  |
| Size enlargement^*^ |  |  |  |  |  |  |
| Absence | Reference | | | Reference |  |  |
| Presence | 9.919 | 4.493, 21.899 | <.001 | 5.905 | 2.086, 16.716 | .001 |
| Margin |  |  |  |  |  |  |
| Circumscribed | Reference | | |  |  |  |
| Non-circumscribed | 3.538 | 1.765, 7.093 | <.001 |  |  |  |
| Shape |  |  |  |  |  |  |
| -Round/Oval | Reference | | |  |  |  |
| Irregular | 4.492 | 2.196, 9.191 | <.001 |  |  |  |
| **Perinodular features** |  |  |  |  |  |  |
| Nourishing vessels |  |  |  |  |  |  |
| Absence | Reference | | |  |  |  |
| Presence | 3.798 | 1.855, 7.779 | <.001 |  |  |  |
| Breast density^^^ |  |  |  |  |  |  |
| nondense | Reference |  |  |  |  |  |
| dense | 1.698 | (0.607,4.749) | .313 |  |  |  |

Note.—AT = arrival time, RT = rise time, TTP = time to peak, MTT = mean transit time, FT = fall time.

^‡^ Menopausal status: Women aged 60 years or older, reporting a history of hysterectomy, or reporting no periods within the past 12 months

without the use of hormonal contraceptives were categorized as postmenopausal. Women reporting regular periods (12–18 times in the last 12

months) without the use of hormonal contraceptives were categorized as premenopausal.

^&^ The wash-in time of breast lesions compared with that of the breast mammary gland.

^*^ The lesion size measured in the contrast-enhanced ultrasound is larger than that in the gray ultrasound.

^^^Breast density assessed with Magnetic Resonance Imaging according to the ACR BI-RADS Magnetic Resonance Imaging categories a, b, c, d. In this study, c and d categories of breast density isare defined as dense breastbreasts. On the other hand, the, a and b categories of breast density isare defined as non-dense breastnon-dense breasts.

**TABLE S8.** Logistic analysis of the MP-MRI model for breast lesions

|  | Univariable | | | Multivariable | | |
| --- | --- | --- | --- | --- | --- | --- |
| Variable | Odds Radio | 95% CI | *P* Value | Odds Radio | 95% CI | *P* Value |
| Age(y) |  |  |  |  |  |  |
| < 49 | Reference |  |  |  |  |  |
| ≧49 | 3.627 | (1.941, 6.780) | <.001 |  |  |  |
| Menopausal status^‡^ |  |  |  |  |  |  |
| Before | Reference |  |  |  |  |  |
| After | 2.699 | 1.412, 5.519 | .003 |  |  |  |
| Nulliparity |  |  |  |  |  |  |
| No | Reference |  |  |  |  |  |
| Yes | 1.698 | 0.607, 4.749 | .313 |  |  |  |
| Diameter(cm) |  |  |  |  |  |  |
| < 1.5 | Reference |  |  |  |  |  |
| ≧1.5 | 3.381 | (1.799, 6.355) | <.001 | 3.844 | (1.637, 9.025) | .002 |
| Type of lesion |  |  |  |  |  |  |
| Focus/foci only | Reference | | |  |  |  |
| Mass | 3.674 | 0.324, 41.697 | .294 |  |  |  |
| Nonmass enhancement | 2.364 | 0.188, 29.707 | .505 |  |  |  |
| No enhancement | 0.000 | － | .999 |  |  |  |
| Shape |  |  |  |  |  |  |
| -Round/Oval | Reference | | |  |  |  |
| Irregular | 2.000 | 0.987, 4.052 | .054 |  |  |  |
| Margin |  |  |  |  |  |  |
| Circumscribed | Reference | | | Reference |  |  |
| Non-circumscribed | 6.431 | 3.102, 13.331 | <.001 | 4.425 | 1.585, 12.349 | .005 |
| Homogeneous |  |  |  |  |  |  |
| Presence | Reference | | |  |  |  |
| Absence | 2.749 | 1.330, 5.682 | .006 |  |  |  |
| Uptake pattern |  |  |  |  |  |  |
| Other | Reference |  |  |  |  |  |
| Centripetal | 1.219 | 0.103, 14.408 | .875 |  |  |  |
| Centrifugal | 0.281 | 0.022, 3.550 | .327 |  |  |  |
| Diffuse | 0.86 | 0.074, 9.971 | .904 |  |  |  |
| Rim enhancement |  |  |  |  |  |  |
| Absence | Reference |  |  |  |  |  |
| Presence | 2.557 | 1.071, 6.105 | .035 |  |  |  |
| Claw-shaped pattern |  |  |  |  |  |  |
| Absence | Reference |  |  |  |  |  |
| Presence | 9.937 | 3.645, 27.096 | <.001 |  |  |  |
| Kinetics—initial phase |  |  |  |  |  |  |
| Slow | Reference | | |  |  |  |
| Medium | 6.243 | 2.267, 17.195 | <.001 |  |  |  |
| Rapid | 5.714 | 2.052, 15.915 | .001 |  |  |  |
| Kinetics—delayed phase |  |  |  |  |  |  |
| Persistent | Reference | | |  |  |  |
| Plateau | 4.641 | 1.798, 11.977 | .002 |  |  |  |
| Washout | 25.071 | 8.214, 76.522 | <.001 |  |  |  |
| T1W |  |  |  |  |  |  |
| High | Reference | | |  |  |  |
| Equal | 0.351 | 0.085, 1.452 | .148 |  |  |  |
| Low | 0.875 | 0.214, 3.586 | .853 |  |  |  |
| T2W |  |  |  |  |  |  |
| Low | Reference |  |  |  |  |  |
| High | 1.128 | 0.499, 2.550 | .772 |  |  |  |
| Equal | 0.827 | 0.288, 2.374 | .724 |  |  |  |
| DWI |  |  |  |  |  |  |
| Other | Reference |  |  | Reference |  |  |
| High | 4.044 | 1.720, 9.508 | .001 | 4.867 | 1.380, 17.170 | .014 |
| ADC value (10^−3^ mm^2^ /s) | 0.017 | 0.005, 0.065 | <.001 | 0.040 | 0.008, 0.211 | <.001 |
| **Perinodular features** |  |  |  |  |  |  |
| Breast density^^^ |  |  |  |  |  |  |
| Nondense | Reference |  |  |  |  |  |
| Dense | 1.698 | (0.607, 4.749) | .313 |  |  |  |
| BPE |  |  |  |  |  |  |
| Minimal | Reference | | | Reference |  |  |
| Mild | 3.543 | 1.159, 10.832 | .027 | 0.273 | 0.071, 1.055 | .060 |
| Moderate | 2.981 | 1.029, 8.639 | .044 | 0.180 | 0.036, 0.893 | .036 |
| Marked | 3.214 | 0.905, 11.411 | .071 | 0.111 | 0.023, 0.538 | .006 |

Note.—BPE = Background Parenchymal Enhancement, DWI = Diffusion Weighted Imaging, ADC = Apparent Diffusion Coefficient.

^‡^ Menopausal status: Women aged 60 years or older, reporting a history of hysterectomy, or reporting no periods within the past 12 months

without the use of hormonal contraceptives were categorized as postmenopausal. Women reporting regular periods (12–18 times in the last 12

months) without the use of hormonal contraceptives were categorized as premenopausal.

^^^Breast density assessed with Magnetic Resonance Imaging according to the ACR BI-RADS Magnetic Resonance Imaging categories a, b, c, d. In this study, c and d categories of breast density isare defined as dense breastbreasts. On the other hand, the, a and b categories of breast density isare defined as non-dense breastnon-dense breasts.

**TABLE S9.** Logistic analysis of hybrid model variables for breast lesions

|  | Univariable | | | Multivariable | | |
| --- | --- | --- | --- | --- | --- | --- |
| Variable | Odds Radio | 95% CI | *P* value | Odds Radio | 95% CI | *P* value |
| Age (y) |  |  |  |  |  |  |
| < 49 | Reference |  |  |  |  |  |
| ≧49 | 4.189 | (1.810, 9.697) | .001 |  |  |  |
| **Intranodular features** |  |  |  |  |  |  |
| Diameter (cm) |  |  |  |  |  |  |
| < 1.5 | Reference |  |  |  |  |  |
| ≧1.5 | 3.381 | (1.285, 6.940) | .011 |  |  |  |
| FT≦14.1 s |  |  |  |  |  |  |
| Absence | Reference |  |  |  |  |  |
| Presence | 9.372 | 3.358, 26.152 | <.001 | 11.386 | 3.913, 33.130 | <.001 |
| Size enlargement^*^ |  |  |  |  |  |  |
| Absence | Reference |  |  |  |  |  |
| Presence | 5.905 | 2.086, 16.716 | .001 | 11.678 | 4.225, 32.281 | <.001 |
| Margin in MP-MRI |  |  |  |  |  |  |
| Circumscribed | Reference |  |  |  |  |  |
| Non-circumscribed | 4.425 | 1.585, 12.349 | .005 |  |  |  |
| DWI |  |  |  |  |  |  |
| Other | Reference |  |  | Reference |  |  |
| High | 4.867 | 1.380, 17.170 | .014 | 5.109 | 1.274, 20.493 | .021 |
| ADC value (10^−3^ mm2/s) | 0.040 | 0.008, 0.211 | <.001 | 0.015 | 0.004, 0.065 | <.001 |
| **Perinodular features** |  |  |  |  |  |  |
| BPE |  |  |  |  |  |  |
| Minimal | Reference |  |  |  |  |  |
| Mild | 0.273 | 0.071, 1.055 | .060 |  |  |  |
| Moderate | 0.180 | 0.036, 0.893 | .036 |  |  |  |
| Marked | 0.111 | 0.023, 0.538 | .006 |  |  |  |

Note.—FT = fall time, BPE = background parenchymal enhancement, DWI=diffusion-weighted imaging, ADC=apparent diffusion coefficient

^*^ The lesion size measured on contrast-enhanced ultrasound was larger than that measured on gray ultrasound.

**TABLE S10.** AUC value for CEUS, MRI and hybrid models in the development cohort and validation cohorts.

|  | Development cohort (n = 151) | *P* Value | Internal validation cohort (n = 151) | | | External validation cohort (n = 35) | *P* Value |
| --- | --- | --- | --- | --- | --- | --- | --- |
|  |  |  | 1000 Bootstrap | 5-time cross | 10-time cross |  |  |
| PFB-CEUS |  |  |  |  |  |  |  |
| Present model | 0.90 (0.84, 0.94) |  | 0.90(0.84, 0.95) | 0.88 | 0.88 | 0.89 (0.74, 0.97) |  |
| BI-RADS | 0.88 (0.82, 0.93) | .64^*^ | NA | NA | NA | 0.80 (0.63, 0.92) | .43^*^ |
| Chen model | 0.71 (0.64, 0.78) | <.001^*^ | NA | NA | NA | 0.69 (0.51, 0.83) | .02^*^ |
| Luo model | 0.68 (0.60, 0.75) | <.001^*^ | NA | NA | NA | 0.74 (0.57, 0.87) | .01^*^ |
| Yukio model | 0.62 (0.054, 0.70) | <.001^*^ | NA | NA | NA | 0.70 (0.53, 0.85) | .01^*^ |
| MP-MRI |  |  |  |  |  |  |  |
| Present model | 0.90 (0.85, 0.95) | .80^*^ | 0.90 (0.85, 0.96) | 0.89 | 0.87 | 0.89 (0.73, 0.97) | .85^*^ |
| BI-RADS | 0.85 (0.73, 0.97) | .10^§^ | NA | NA | NA | 0.73 (0.55, 0.87) | .15^§^ |
| Hybrid |  |  |  |  |  |  |  |
| Present model | 0.95 (0.90, 0.98) | .01^*^  .08^§^ | 0.95 (0.91, 0.99) | 0.94 | 0.94 | 0.92 (0.77, 0.98) | .29^*^  .40^§^ |

Note.—Data in parentheses are 95% confidence intervals. NA means the model’s breast lesion diagnosis in the internal validation cohort is absent.

^*^ *P* value for comparisons with the present PFB-CEUS model.

^§^ *P* value for comparisons with the present MP-MRI model.

**TABLE S11.** AUCs of PFB-CEUS, MP-MRI, and hybrid models in subgroups of patients

|  | Development cohort | *P* value | Validation cohort | *P* value |
| --- | --- | --- | --- | --- |
| Age (y) |  |  |  |  |
| < 49 |  |  |  |  |
| PFB-CEUS | 0.86 (0.76, 0.93) |  | 0.87 (0.58, 0.99) |  |
| MP-MRI | 0.90 (0.81, 0.96) | 0.554^*^ | 0.94 (0.67, 1.00) | 0.559^*^ |
| Hybrid | 0.96 (0.89, 0.99) | 0.005^*^ | 0.94 (0.67, 1.00) | 0.205^*^ |
|  |  | 0.148^§^ |  | 1.000^§^ |
| ≧49 |  |  |  |  |
| PFB-CEUS | 0.87 (0.77, 0.94) |  | 0.89 (0.67, 0.98) |  |
| MP-MRI | 0.85 (0.74, 0.92) | 0.618^*^ | 0.85 (0.62, 0.97) | 0.341^*^ |
| Hybrid | 0.91 (0.83, 0.97) | 0.153^*^ | 0.89 (0.67, 0.98) | 1.000^*^ |
|  |  | 0.151^§^ |  | 0.318^§^ |
| Menstrual status |  |  |  |  |
| Premenopausal |  |  |  |  |
| PFB-CEUS | 0.86 (0.77, 0.92) |  | 0.91 (0.69, 0.99) |  |
| MP-MRI | 0.89 (0.81, 0.95) | 0.491^*^ | 0.91 (0.69, 0.99) | 1.000^*^ |
| Hybrid | 0.94 (0.87, 0.98) | 0.006^*^ | 0.94 (0.74, 1.00) | 0.231^*^ |
|  |  | 0.214^§^ |  | 0.646^§^ |
| Postmenopausal |  |  |  |  |
| PFB-CEUS | 0.93 (0.83, 0.98) |  | 0.80 (0.53, 0.96) |  |
| MP-MRI | 0.89 (0.78, 0.96) | 0.374^*^ | 0.79 (0.52, 0.95) | 0.841^*^ |
| Hybrid | 0.97 (0.89, 1.00) | 0.181 ^*^ | 0.81 (0.54, 0.96) | 0.760^*^ |
|  |  | 0.101^§^ |  | 0.625^§^ |

^*^*P* value for comparisons with the present PFB-CEUS model.

^§^ *P* value for comparisons with the present MP-MRI model.

**TABLE S12.** Diagnostic results of PFB-CEUS, MP-MRI, and hybrid models in high-risk lesions.

| Histologic type | BI-RADS of MRI on-site | BI-RADS of MRI review | | BI-RADS of CEUS on-site | BI-RADS of CEUS review | Malignant Prob of CEUS model | Malignant Prob of MRI model | Malignant Prob of Hybrid model |
| --- | --- | --- | --- | --- | --- | --- | --- | --- |
| intraductal papilloma | 4b | 4b | 3 | | 4a | 0.0552 | 0.0001 | 0.1174 |
| intraductal papilloma | 4c | 4C | 4c | | 4b | 0.1252 | 0.3220 | 0.2555 |
| intraductal papilloma | 4c | 4C | 4c | | 4b | 0.1252 | 0.3220 | 0.2555 |
| intraductal papilloma | 4b | 4C | 4b | | 4b | 0.1639 | 0.0482 | 0.1056 |
| intraductal papilloma | 4b | 4C | 4b | | 4b | 0.1639 | 0.0482 | 0.1056 |
| intraductal papilloma | 5 | 4a | 4c | | 5 | 0.2031 | 0.0462 | 0.0127 |
| intraductal papilloma | 3 | 3 | 3 | | 3 | 0.6701 | 0.2808 | 0.7394 |
| intraductal papilloma | 3 | 3 | 3 | | 3 | 0.6701 | 0.4065 | 0.4680 |
| intraductal papilloma | 4b | 4b | 4b | | 5 | 0.8531 | 0.9807 | 0.8486 |
| phyllodes tumor | 3 | 3 | 4a | | 3 | 0.4583 | 0.1731 | 0.2223 |

**TABLE S13.** False-positive and false-negative correction rates of the three models in different radiologists’ diagnostic results

|  | BI-RADS (3 vs. 4a+) | | BI-RADS (3, 4a vs. 4b+) | | BI-RADS (3, 4a, 4b vs. 4c+) | |
| --- | --- | --- | --- | --- | --- | --- |
|  | FPCR (%) | FNCR (%) | FPCR (%) | FNCR (%) | FPCR (%) | FNCR (%) |
| PFB-CEUS on-site^*^ |  |  |  |  |  |  |
| CEUS model | 80.6 (25/31) | 66.7 (2/3) | 73.3 (11/15) | 85.7 (6/7) | 100.0 (6/6) | 80.0 (20/25) |
| Hybrid model | 90.3 (28/31) | 66.7 (2/3) | 93.3 (14/15) | 71.4 (5/7) | 100.0 (6/6) | 72.0 (18/25) |
| PFB-CEUS reviewers^†^ |  |  |  |  |  |  |
| CEUS model | 82.2 (37/45) | 83.3 (5/6) | 66.7 (14/21) | 68.7 (11/16) | 66.7 (10/15) | 72.0 (18/25) |
| Hybrid model | 88.9 (40/45) | 83.3 (5/6) | 76.1 (16/21) | 75.0 (12/16) | 66.7 (10/15) | 72.0 (18/25) |
| MP-MRI on-site^*^ |  |  |  |  |  |  |
| MRI model | 77.7 (28/36) | 50.0 (1/2) | 73.6 (14/19) | 44.4 (4/9) | 66.7 (6/9) | 60.8 (14/23) |
| Hybrid model | 86.1 (31/36) | 0.0 (0/0) | 89.4 (17/19) | 66.7 (6/9) | 88.9 (8/9) | 73.9 (17/23) |
| MP-MRI reviewers^†^ |  |  |  |  |  |  |
| MRI model | 83.0 (44/53) | 57.1 (4/7) | 81.5 (31/38) | 58.3 (7/12) | 78.2 (18/23) | 71.4 (15/21) |
| Hybrid model | 90.5 (48/53) | 42.8 (3/7) | 89.4 (34/38) | 50.0 (6/12) | 86.9 (20/23) | 71.4 (15/21) |

Note.—FPCR = false-positive correction rate, FNCR = false-negative correction rate.

* 25 radiologists who performed PFB-CEUS and 27 radiologists who performed MP-MRI.

† 3 radiologists who reviewed the stored PFB-CEUS and 3 radiologists who reviewed the stored MP-MRI images.

**References**

1. Du J, Wang L, Wan C-F et al. Differentiating benign from malignant solid breast lesions: combined utility of conventional ultrasound and contrast-enhanced ultrasound in comparison with magnetic resonance imaging. Eur J Radiol 2012;81(12):3890-3899

2. Miyamoto Y, Ito T, Takada E et al. Efficacy of Sonazoid (perflubutane) for contrast-enhanced ultrasound in the differentiation of focal breast lesions: phase 3 multicenter clinical trial. AJR 2014;202(4):W400-W407

3. Pan J, Tong W, Luo J et al. Does contrast-enhanced ultrasound (CEUS) play a better role in diagnosis of breast lesions with calcification? A comparison with MRI. Br J Radiol 2020;93(1112):20200195

4. Li C, Yao M, Shao S et al. Diagnostic efficacy of contrast-enhanced ultrasound for breast lesions of different sizes: a comparative study with magnetic resonance imaging. Br J Radiol 2020;93(1110):20190932

5. Liu W, Zong M, Gong H-Y et al. Comparison of diagnostic efficacy between contrast-enhanced ultrasound and DCE-MRI for mass- and non-mass-like enhancement types in breast lesions. Cancer Manag Res 2020;12:13567-13578


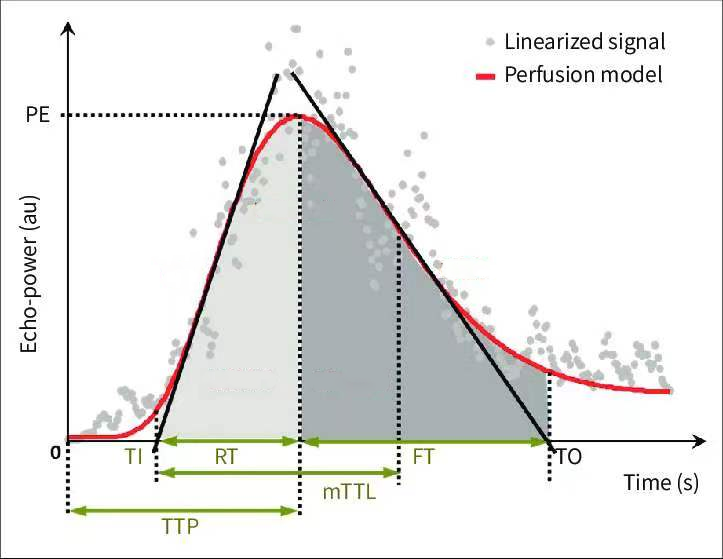
**
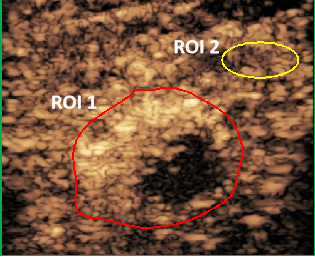
**

**
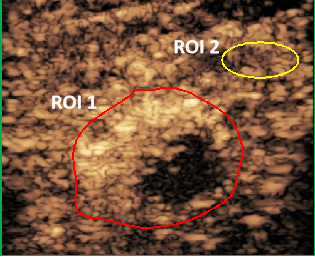
**

**FIGURE S1.** Time-intensity curve analysis according to region of interest (ROI) on contrast-enhanced ultrasound. The FT, RT, TTP, mTT, AT in the time intensity curve of the PFB-CEUS. FT = Fall Time, RT = Rise Time, TTP = Time To Peak, mTT = mean Transit Time, AT= Arrival time, PFB-CEUS = Perfluorobutane-Contrast enhanced Ultrasound.


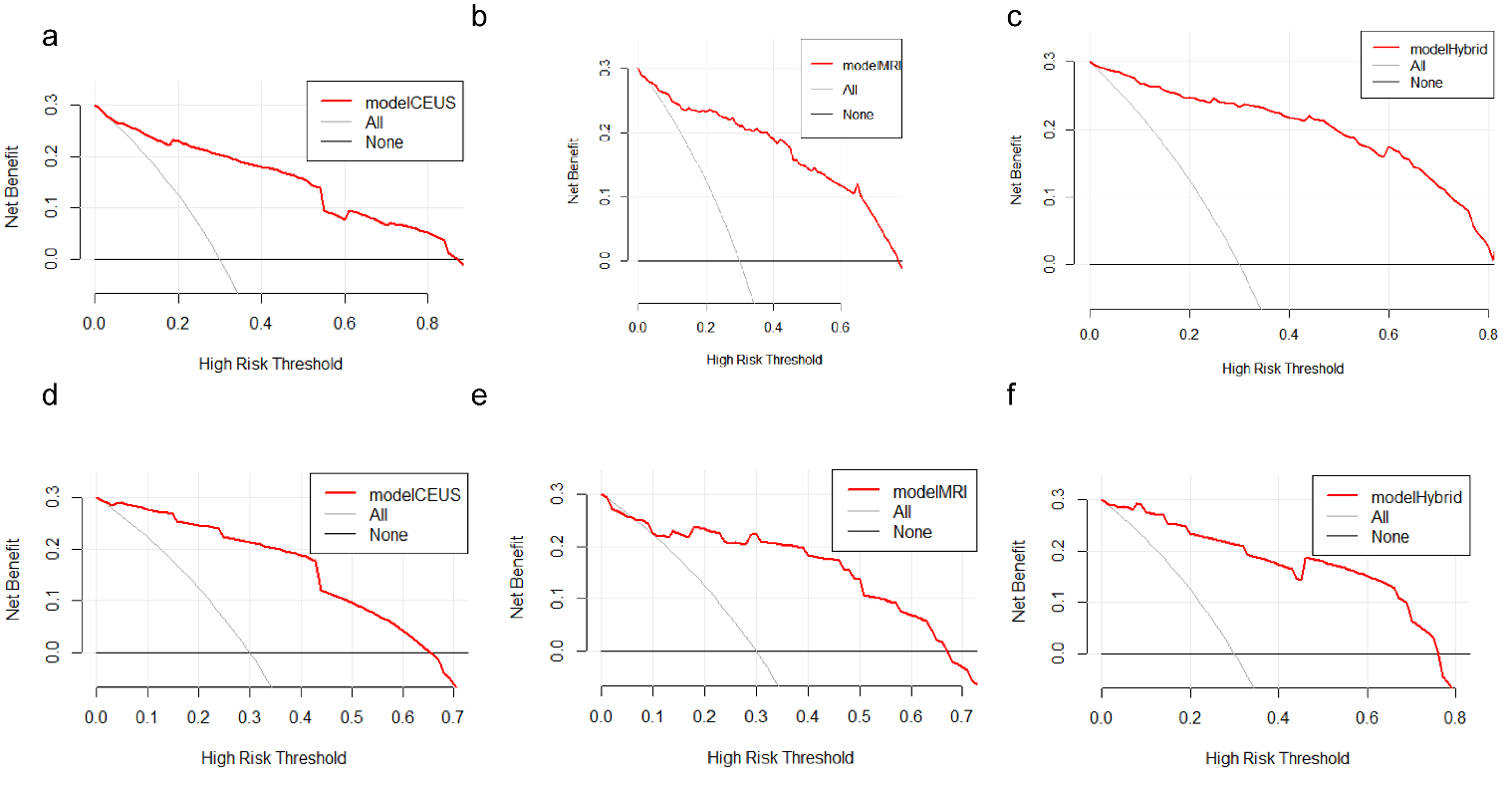


**FIGURE S2.** Decision curve analysis for PFB-CEUS MP-MRI, , and hybrid models in the development and external validation cohort. (A-C) PFB-CEUS, MP-MRI, and hybrid models in the development cohort. (D-F) PFB-CEUS, MP-MRI, and hybrid models in the external validation cohort. MP-MRI =Multiparametric-Magnetic Resonance Imaging, PFB-CEUS = Perfluorobutane-Contrast enhanced Ultrasound.


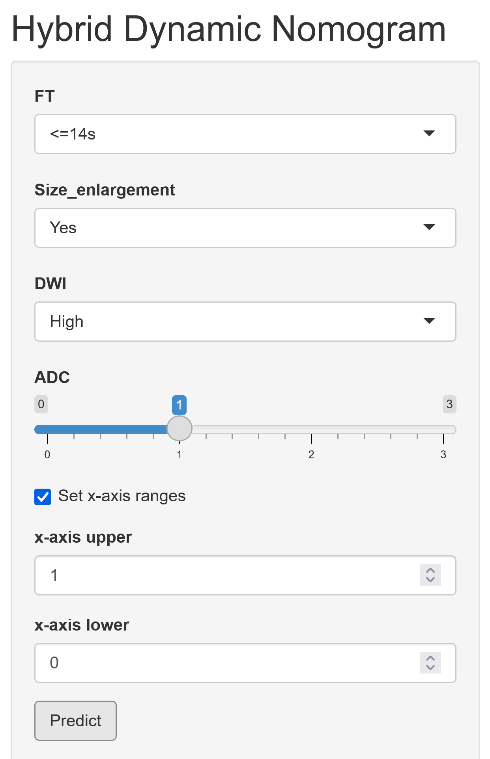


**FIGURE S3 .** The hybrid model nomogram for a 67-year-old woman with an invasive carcinoma by pathological diagnosis.


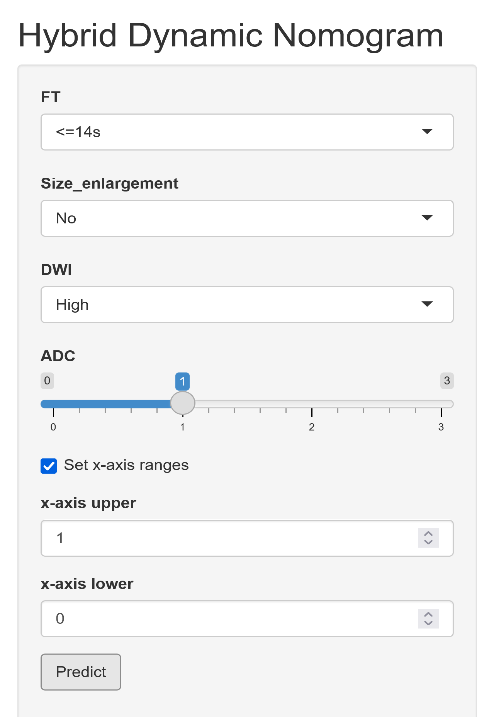


**FIGURE S4 .** The hybrid model nomogram for a 50-year-old woman with an invasive carcinoma by pathological diagnosis.

**Supplementary Materials and Methods S1: Patient Recruitment**

The inclusion criteria were (a) patients with breast lesions newly diagnosed with conventional US screening as Breast Imaging-Reporting and Data System (BI-RADS) categories 3, 4, and 5; (b) MP-MRI performed at a 30-day interval with PFB-CEUS; and (c) no contraindications for puncture biopsy, with histopathological examination obtained for each target lesion.

The exclusion criteria were (a) contraindications to PFB-CEUS or MP-MRI (e.g., patients who had an egg or milk allergy, were pregnant or lactating, or had renal dysfunction, etc.), (b) patients who had received previous treatment for the target lesions, including systemic therapy or chest radiotherapy, or (c) unclear pathologic findings.

**Supplementary Materials and Methods S2: Steps of bootstrapping**

1. Construct a model in the original sample; determine the apparent performance on the data from the sample used to construct the model;

2. Draw a bootstrap sample with replacement from the original sample;

3. Construct a model (Model*) in Sample*, repeating every step that was performed in the original sample, especially model specification steps such as selection of predictors. Determine the bootstrap performance as the apparent performance of Model* in Sample*;

4. Apply Model* to the original sample without any modification to determine the test performance;

5. Calculate the optimism as the difference between bootstrap performance and test performance;

6. Repeat steps 1–4 many times (at least 200) to obtain an sTABLE mean estimate of optimism;

7. Subtract the mean optimism estimate (step 6) from the apparent performance (step 1) to obtain the optimism-corrected performance estimate. (1)

**Supplementary Materials and Methods S3: Operating Point Selection**

Three sets of sensitivity and specificity values were calculated: (a) a BI-RADS assessment of category 4A and higher (i.e., 4A, 4B, 4C, and 5) defined a positive call for cancer diagnosis, and conversely, a BI-RADS assessment of category 3 defined a negative call for cancer diagnosis; (b) a BI-RADS assessment of category 4B and higher (4B, 4C, and 5) defined a positive call, and conversely, a BI-RADS assessment of categories 3 and 4A defined a negative call; (c) a BI-RADS assessment of category 4C and higher (4C and 5) defined a positive call, and conversely, a BI-RADS assessment of categories 3, 4A, and 4B defined a negative call. The models’ false-positive identified rate (FPCR) and false-negative identified rate (FNCR) were also reported.

The performance of different models was compared by detecting differences in the AUCs using Delong’s test. The calibration of the model was accompanied by the Hosmer‒Lemeshow test. The models were validated using the validation cohort. Age group (< 49 years and ≧49 years) and menstrual status (premenopausal or postmenopausal) were stratified for further analysis to detect possible differences between the training cohort and validation cohort.

**Supplementary Materials and Methods S4: Methods of Re-CEUS BI-RADS**

A standardized lexicon or terminology system for CEUS does not yet exist, similar to the ACR MRI BI-RADS lexicon. In the last decade, radiologist-scored CEUS BI-RADS[2,3] was determined according to the rules specified in a previous study called “rerated BI-RADS”[4]. The specific step of rerated BI-RADS was at this step:

In the first step, based on the enhancement characteristic in the CEUS of the lesions, the CEUS was scored as a 5-point system.

| CEUS Score | Enhancement Patterns |
| --- | --- |
| 1 | There is no enhancement in the lesion, and a clear borderline separates the lesion from the surrounding tissue. |
| 2 | There is iso- and synchronous enhancement with the surrounding tissue, without a clear outline in the contrast-enhanced image. |
| 3 | The lesion exhibits earlier enhancement compared with the surrounding tissue, homogeneous or heterogeneous, with a clear margin (sometimes with ring like enhancement). The lesion is almost identical in size to or smaller than that in a 2-D image. The shape of the lesion is regular: round or oval. |
| 4 | The lesion exhibits earlier enhancement than the surrounding tissue, usually heterogeneous. The lesion in the contrast-enhanced image is larger than that in the corresponding 2-D image, but still has a clear margin, with or without a perfusion defect in the lesion and without crab claw-like enhancement. The shape of the lesion is always irregular. |
| 5 | The lesion is heterogeneously enhanced, with a larger scope than that of the corresponding 2-D image. There is earlier enhancement, a perfusion defect may be present or absent and usually a typical crab claw-like enhancement with an unclear margin is evident. |

Second, CEUS was used to rerate ACR ultrasound BI-RADS [5] categories via the rules specified in the following table.

| Original BI-RADS category | CEUS Score | Modified BI-RADS category |
| --- | --- | --- |
| 3, 4a | 1-3 | Remains unchanged |
|  | 4 | Increase one level |
|  | 5 | Increase two levels |
| 4b, 4c | 1-2 | Reduce two levels |
|  | 3 | Reduce one level |
|  | 4-5 | Increase one level |
| 5 | 1-3 | Reduce one level |
|  | 4-5 | Remains unchanged |

References

[1]. Clinical Prediction Models A Practical Approach to Development,Validation, and Updating. 2 ed. Springers Nature, 2019.

[2]. Pan J, Tong W, Luo J, Liang J, Pan F, Zheng Y, et al. Does contrast-enhanced ultrasound (CEUS) play a better role in diagnosis of breast lesions with calcification? A comparison with MRI. Br J Radiol. 2020;93:20200195.

[3]. Li C, Yao M, Shao S, Li X, Li G, Wu R. Diagnostic efficacy of contrast-enhanced ultrasound for breast lesions of different sizes: a comparative study with magnetic resonance imaging. Br J Radiol. 2020;93:20190932.

[4]. Xiao X, Dong L, Jiang Q, Guan X, Wu H, Luo B. Incorporating Contrast-Enhanced Ultrasound into the BI-RADS Scoring System Improves Accuracy in Breast Tumor Diagnosis: A Preliminary Study in China. Ultrasound Med Biol. 2016 Nov;42(11):2630-2638. doi: 10.1016/j.ultrasmedbio.2016.07.005. Epub 2016 Aug 17. PMID: 27544439.

[5]. Mendelson EB, Böhm-Vélez M, Berg WA, et al. ACR BI-RADS® Ultrasound. In: ACR BI-RADS® Atlas, Breast Imaging Reporting and Data System. Reston, VA, American College of Radiology; 2013
